# Fine-mapping analysis revealed complex pleiotropic effect and tissue-specific regulatory mechanism of TNFSF15 in primary biliary cholangitis, Crohn’s disease and leprosy

**DOI:** 10.1038/srep31429

**Published:** 2016-08-10

**Authors:** Yonghu Sun, Astrid Irwanto, Licht Toyo-oka, Myunghee Hong, Hong Liu, Anand Kumar Andiappan, Hyunchul Choi, Yuki Hitomi, Gongqi Yu, Yongxiang Yu, Fangfang Bao, Chuan Wang, Xian Fu, Zhenhua Yue, Honglei Wang, Huimin Zhang, Minae Kawashima, Kaname Kojima, Masao Nagasaki, Minoru Nakamura, Suk-Kyun Yang, Byong Duk Ye, Yosua Denise, Olaf Rotzschke, Kyuyoung Song, Katsushi Tokunaga, Furen Zhang, Jianjun Liu

**Affiliations:** 1 Shandong Provincial Institute of Dermatology and Venereology, Shandong Academy of Medical Sciences, Jinan, Shandong 250000, China; 2 Shandong Provincial Key Laboratory for Dermatovenereology, Jinan, Shandong 250000, China; 3 Shandong Provincial Hospital for Skin Diseases, Shandong University, Jinan, Shandong 250000, China; 4 Human Genetics, Genome Institute of Singapore, Singapore 138672, Singapore; 5 Human Genetics, University of Tokyo, Graduate School of Medicine, Tokyo 113-0033, Japan; 6 Biochemistry and Molecular Biology, University of Ulsan College of Medicine, Seoul 138736, Korea; 7 Immune Regulation, Singapore Immunology Network, Singapore 138648, Singapore; 8 Integrative Genomics, Tohoku Medical Megabank Organization, Tohoku University, Sendai, 980-8573, Japan; 9 Clinical Research Center, National Hospital Organization Nagasaki Medical Center, Onuma, Nagasaki 856-8562, Japan; 10 Gastroenterology, Asan Medical Center, University of Ulsan College of Medicine, Seoul 138736, Korea; 11 Faculty of Biotechnology, Atma Jaya Catholic University of Indonesia, South Jakarta 12930, Indonesia; 12 School of Medicine, Shandong University, Jinan, Shandong 250000, China; 13 National Clinical Key Project of Dermatology and Venereology, Jinan, Shandong 250000, China

## Abstract

Genetic polymorphism within the 9q32 locus is linked with increased risk of several diseases, including Crohn’s disease (CD), primary biliary cholangitis (PBC) and leprosy. The most likely disease-causing gene within 9q32 is *TNFSF15*, which encodes the pro-inflammatory cytokine TNF super-family member 15, but it was unknown whether these disparate diseases were associated with the same genetic variance in 9q32, and how variance within this locus might contribute to pathology. Using genetic data from published studies on CD, PBC and leprosy we revealed that bearing a T allele at rs6478108/rs6478109 (r^2^ = 1) or rs4979462 was significantly associated with increased risk of CD and decreased risk of leprosy, while the T allele at rs4979462 was associated with significantly increased risk of PBC. *In vitro* analyses showed that the rs6478109 genotype significantly affected *TNFSF15* expression in cells from whole blood of controls, while functional annotation using publicly-available data revealed the broad cell type/tissue-specific regulatory potential of variance at rs6478109 or rs4979462. In summary, we provide evidence that variance within *TNFSF15* has the potential to affect cytokine expression across a range of tissues and thereby contribute to protection from infectious diseases such as leprosy, while increasing the risk of immune-mediated diseases including CD and PBC.

Previous genome-wide association studies (GWAS) have revealed multiple associations of diverse disease phenotypes on chromosome 9q32, including primary biliary cholangitis (previously known as primary biliary cirrhosis^1^,^2^,^3^,^4^,^5^,^6^,^7^,^8^)(PBC)^9^, Crohn’s disease (CD)^10^,^11^,^12^,^13^, and leprosy^14^. However, the identified associations seem to show opposite effects, increasing risk of PBC and CD, while decreasing risk of leprosy^15^. Among the genes within the critical region of the locus, *TNFSF15* has been identified as the likely candidate mediating the observed disease-associations^16^. TNFSF15 (also named TL1A, tumor necrosis factor superfamily member 15) is a newly-described cytokine that promotes T cell proliferation^17^, augments production of T-helper 1 (Th1) cytokines (in particular interferon gamma (IFNγ)^18^, and promotes Th17 effector cell function^19^. There is accumulating evidence for a role of *TNFSF15* in infectious and autoimmune disease development^20^: both TNFSF15 and its receptor (DcR3) are more abundant in sera from PBC patients compared to healthy controls^21^,^22^; while *TNFSF15* expression was significantly higher in biopsies from inflamed CD lesions than those from uninvolved tissues. A study showing that blockade of TNFSF15 attenuates intestinal inflammation in inflammatory bowel disease^23^ highlights the potential impact of overexpression of this potent cytokine.

While PBC, CD and leprosy share some pathological features, such as granuloma formation^24^, the different effects of variance at 9q32 upon risk of these diseases, and the discovery of these risk variants in specific populations, mean that important questions on the overall role of 9q32 polymorphisms, specifically those affecting *TNFSF15*, remain unanswered. To resolve this situation we performed an integrated fine-mapping study of the *TNFSF15* locus using published genetic data from PBC patients and controls in a Japanese population, CD patients and controls in a Korean population, and leprosy patients and controls in a Chinese population. We identified and ranked single-nucleotide polymorphisms (SNP) associated with each disease and used *in silico* functional annotation combined with *in vitro* experimentation to show that the diverse disease associations within *TNFSF15* are probably driven by two independent causal variants that contribute to regulating *TNFSF15* expression across a range of tissues and cell types.

## Results

### Fine mapping analysis of the *TNFSF15* locus in Leprosy, CD and PBC by imputation

We selected several previously-published GWAS datasets for fine-mapping analysis of the *TNFSF15* locus: two studies of leprosy in Chinese ethnicity populations (totalling 9,619 cases and 12,896 controls)^14^,^15^, two studies of CD in Korean ethnicity populations (totalling 1,575 cases and 3,013 controls)^10^,^11^, and a study of PBC in a Japanese ethnicity population (1,594 cases and 1,529 controls)^9^. As these studies did not focus on the 9q32 region specifically, we increased the resolution of data for the locus by imputation using reference panels from the 1000 Genomes Project as well as some population-based sequence data (see Supplementary Methods). After imputation and quality control we identified insertion-deletion (indel) and single-nucleotide polymorphism (SNP) variants (both for genotyped and imputed haplotypes) within the region encompassing 117.25–117.72 Mb on chromosome 9. There were 87.4% and 87.9% of variants with minor allele frequency of equal or above 1% and 5%, respectively, in the 1000 Genomes Project Asian-ancestry haplotypes that were either genotyped or imputed successfully for association analysis.

The association analysis identified strong associations between specific variants and each disease, with evidence surpassing genome-wide significance (Table 1). However, the most significantly associated variants were different SNPs in each case (Figure S1): for Japanese PBC, the most significant SNP was rs55768522, which is in perfect linkage disequilibrium (LD) (r^2^ = 1) with the previously reported SNP, rs4979462^9^; for Korean CD, the most significant SNP was rs6478108, which is in perfect LD with the previously reported rs6478109; while for leprosy, the most significant SNP is rs4366152, which was in high LD with the previously reported SNP, rs6478108 (r^2^ ≥ 0.97, Figure S2). The top SNPs in the Korean CD (rs6478108) and Chinese leprosy (rs4366152) are also in high LD with each other (r^2^ ≥ 0.97), but less so with the top index SNP (rs4979462) of the Japanese PBC (r^2^ ≤ 0.55). Consistently, both rs6478108 and rs4366152 showed strong associations with CD and leprosy, and conditioning on either SNP abolished the extensive associations within the locus in the two disease datasets (Table 1, Figure S3). In the PBC dataset, both rs6478108 and rs4366152 showed much weaker association than rs4979462 (Table 1), and the extensive associations within the locus were abolished by conditioning on rs4979462, but not on rs6478108 or rs4366152 (Figure S4).

In accordance with previous reports^9^,^10^,^11^,^12^,^13^,^14^, for both rs6478108 and rs4979462, T was the risk allele for PBC and CD, but protective for leprosy (Table 1). The haplotype analysis of rs6478108 and rs4979462 revealed consistent results, demonstrating that the common TT haplotype associated with high risk of PBC (OR = 1.68) and CD (OR = 2.57), but low risk of leprosy (OR = 0.68); in contrast the CC haplotype was associated with decreased risk of PBC and CD, but increased risk of leprosy (Table S1).

These results suggest that the opposite associations of CD and leprosy with genetic polymorphism in the *TNFSF15* locus may be driven by the same risk variant, with a different variant in the same gene associated with risk of PBC.

### Replication analysis in independent cohorts of leprosy, CD and PBC

While imputation of absent data from GWAS studies is a robust and valuable tool, it has the potential to increase analytical errors. Therefore, to confirm the results from the fine mapping analysis above, we genotyped the top five most significantly-associated SNPs from the analysis of published leprosy data (including both rs6478108 and rs4366152) and the most significantly-associated SNP from the analysis of published PBC data, rs4979462, in an independent sample of 2,812 leprosy cases and 5,464 controls from China (Table S2). This approach confirmed that rs6478108 was the top index SNP for association with leprosy in Chinese ethnicity individuals (OR = 0.74, P = 2.73 × 10^−26^). Therefore, both leprosy and CD risk associations map to the same SNP, and are likely driven by the same risk variant within the locus.

**Figure 1:**
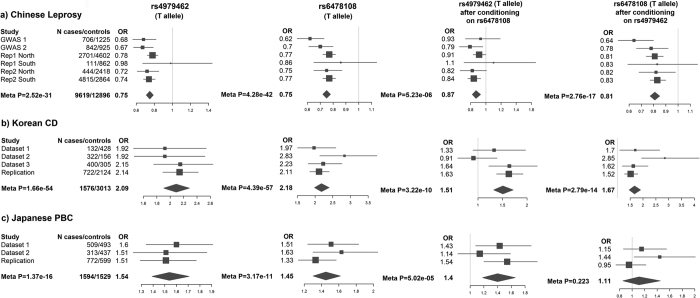
Unconditional and conditional association results of rs4979462 and rs6478108 in the GWAS and replication datasets. Odds ratios are calculated for the A allele of both rs4979462 and rs6478108.

We then genotyped rs6478108 (the top SNP for CD and Leprosy) and rs4979462 (top SNP for PBC) in three independent populations: 5,259 leprosy cases and 5,282 controls of Chinese ethnicity (independent from the initial validation sample above), 722 CD cases and 2,124 controls of Korean ethnicity, and 772 PBC cases and 599 controls of Japanese ethnicity. The analysis of the independent validation samples (totalling 8,071 cases and 10,746 controls for leprosy, 722 cases and 2,124 controls for CD, and 772 cases and 599 controls for PBC) and the joint analysis of the combined GWAS and validation samples of the three diseases (9,619 cases and 12,896 controls for leprosy, 1,576 cases and 3,013 controls for CD, and 1,594 cases and 1,529 controls for PBC) provided strong supportive evidence for the associations of rs6478108 and rs4979462 with risk of the three diseases (Fig. 1).

Interestingly, rs6478108 and rs4979462 showed independent effects on risk of CD and leprosy (Fig. 1): conditioning on rs4979462 reduced the significance of the association at rs6478108, but rs6478108 remained genome-wide significant in both leprosy (P_conditional_ = 2.76 × 10^−17^, OR_conditional_ = 0.81) and CD (P_conditional_ = 2.79 × 10^−14^, OR_conditional_ = 1.67) datasets. Conditioning on rs6478108 also reduced the strength of association at rs4979462 in both leprosy and CD, but the association remained genome-wide significant in CD (P = 3.22 × 10^−10^, OR = 1.51), while becoming suggestive (P_conditional_ = 5.23 × 10^−6^) with significantly reduced OR (from 0.75 to 0.87) in leprosy after conditioning. In contrast, in PBC, conditioning on rs4979462 abolished the risk association at rs6478108 (P_conditional_ = 0.223, OR_conditional_ = 1.11), but not vice versa (rs4979462, P_conditional_ = 5.02 × 10^−5^, OR_conditional_ = 1.4 after conditioning on rs6478108). Taken together, these results indicate that variance at rs6478108 and rs4979462 has independent effects on risk of CD, and possibly of leprosy, but not in the Japanese PBC dataset where rs4979462 remains the top SNP within the *TNFSF15* locus.

**Figure 2:**
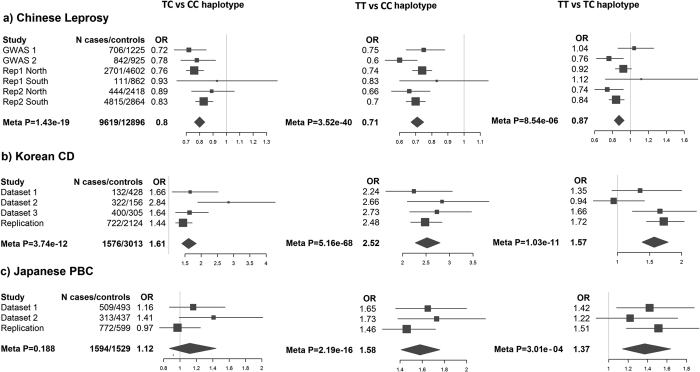
Association results of rs6478108-rs4979462 haplotypes in all three diseases. Reference haplotype from left to right are CC haplotype, CC haplotype and TC haplotype.

The results from the haplotype analysis of rs6478108 and rs4979462 were also consistent with the conditional analysis (Fig. 2). As observed in the GWAS discovery analysis, there were two common haplotypes (TT and CC) and one minor haplotype (TC) in all three populations: in the Korean CD samples, both TT and TC showed significant risk effect (with CC as reference), but the risk effect of TT (OR = 2.52) was 1.6 times higher than that of TC (OR = 1.61), and this difference in risk effect reached genome-wide significance (P = 1.03 × 10^−11^, Fig. 2). Therefore rs6478108_T and rs4979462_T alleles carry independent risk effects. In the Japanese PBC samples, the TT haplotype showed significant risk effect (P = 2.19 × 10^−16^, OR = 1.58), but the TC haplotype did not (P = 0.188, OR = 1.12), though the samples had sufficient power (>80%) to detect a moderate risk effect of the TC haplotype (OR = 1.30) at a P value of 0.05. Thus rs6478108_T is not an independent risk allele (beyond the rs4979462_T allele). In the Chinese leprosy samples, both TC and TT haplotypes were associated with significantly protective effect than the CC haplotype (P = 1.43 × 10^−19^ and P = 3.52 × 10^−40^, respectively) (Fig. 2). The effect of the TC haplotype (OR = 0.80) was approximately 10% less than that of the TT haplotype (OR = 0.71), but this difference did not reach genome-wide significance (P = 8.54 × 10^−6^) (Fig. 2). As for the Korean CD samples, the Chinese leprosy samples did have sufficient power (>80%) to detect the difference between the protective effects of TT and TC at genome-wide significance (Table S3). This suggests that the rs4979462_T allele has independent, but moderate protective effect on risk of leprosy.

### Functional annotation of candidate variants within the *TNFSF15* locus

After dissecting the pattern of genetic associations within the *TNFSF15* locus in CD, leprosy and PBC, we used functional annotation to identify the most likely potential causal SNP(s). Since the two index SNPs, rs6478108 and rs4979462, are located within a LD block that contain multiple variants in high LD, we therefore identified all the SNPs and indels that were tagged by them at r^2^ ≥ 0.8, then carried out functional annotation of these SNPs; we predicted their potential effects on RNA splicing, deoxyribonuclease (DNase) activity, histone modifications, RNA expression (RNA-Seq), and transcription factor (TF) binding (ChIP-Seq) peaks, using data generated in 131 different tissues/cell types from the ENCODE and ROADMAP databases. Alongside, we predicted their impact on expression quantitative trait loci (eQTL) in different blood cells and using published results from functional assays (see Methods).

**Figure 3:**
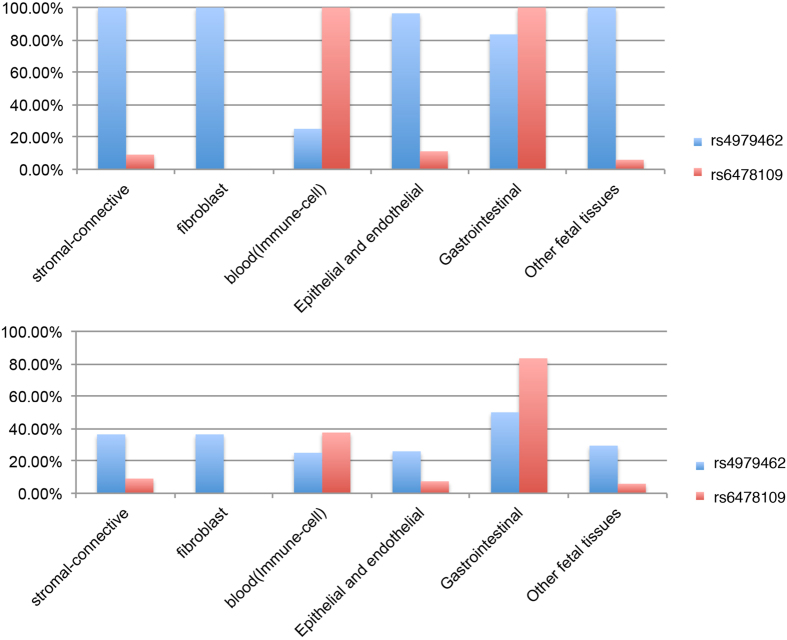
Overlap of peaks of transcriptional regulation marker with rs4979462 and rs6478109 in 81 tissues/cell types. The positive overlapped peaks with DNase hypersensitivity sites (DNase) [Panel A] and histone markers (HM) [Panel B] of rs4979462 and rs6478109. Histone markers include H3K27me3, H3K9me3, H3K27ac, H3K4me1, H3K4me3, and H3K36me3 modifications. Percentage was calculated from the counts of positive peaks upon the total number of each tissue/ cell type category (for more detail see Supplementary Table 5).

Eighteen non-coding SNPs, but no indels, were in high LD with the two index SNPs; of the 18, 6 were within the introns of *TNFSF15,* while the remaining 12 were located adjacent to *TNFSF15* gene (Table S4). None of the 6 intronic SNPs were predicted to affect splicing of *TNFSF15* mRNA. Of the 18 SNPs, five SNPs (rs4263839, rs4979462, rs6478109, rs7848647 and rs56211063) overlapped with DNase peaks in at least one out of 81 cell types (Table S5, Figure S5), suggesting the potential for a regulatory role. After grouping the 81 cell types into eight major categories of cell/tissue types, we examined the predicted impact of these five SNPs on histone modifications, RNA expression and TF binding (Figures S6–S13). Out of the five SNPs, only rs4979462 and rs6478109 overlapped with DNase and histone modification peaks in multiple cell lines (Table S5, Fig. 3), indicating that these two SNPs are the most likely to contribute to regulation of *TNFSF15* expression. Of note, while rs6478109 is in perfect LD with rs6478108, the index SNP for both leprosy and CD, rs4979462 is the index SNP for PBC in the current study.

The two index SNPs also exhibited distinct patterns of predicted regulatory functions across the tissue panels tested: rs4979462 predominantly overlapped DNAse sensitivity sites, histone modification peaks, and enhancers (positive H3K4me1 and H3K27ac peaks) present in stromal and connective tissues, lung and skin fibroblasts, epithelial cancers, epithelial and endothelial cells, heart, muscle, brain and fetal tissues; while rs6478109 predominantly overlapped with DNAse peaks in various immune cells, including primary CD3^+^ cells, CD20^+^ B-cells, Jurkat T-cells, Th-1 T-cells and B-lymphocytes, as well as overlapping an active promoter site of *TNFSF15* (positive H3Kme3 and H3K27ac) in CD14^+^ monocytes (Figure S7). Interestingly, both rs6478109 and rs4979462 were associated with enhancer and promoter markers in gastrointestinal tissues, including stomach, gastric, oesophageal, and small and large intestines. rs6478109-related regulatory sequences in lymphoblastoid cell lines are enriched for the binding sites of TFs like TCF12, RUNX3, IKZF1, POUF2, whereas rs4979462-related regulatory sequences in HeLa-S3 and cervical adenocarcinoma cell lines are enriched for the TF bindings of CEBPB and TCF7L2 (Table S5, Figure S5), suggesting that the two SNPs may interrupt different regulatory processes mediated by different TFs.

**Figure 4:**
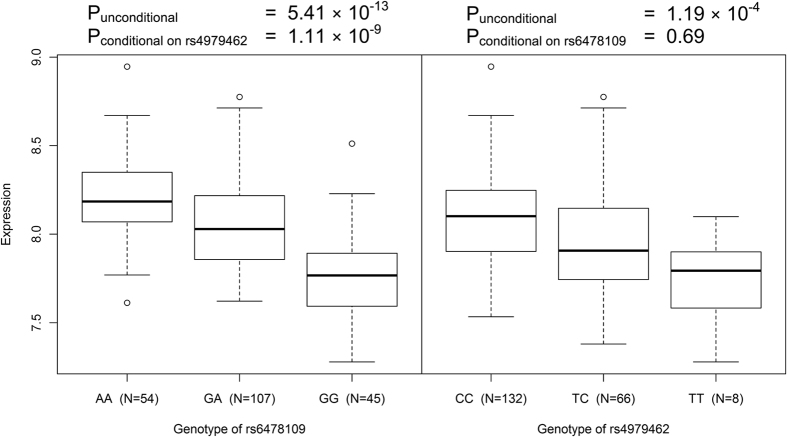
*TNFSF15* mRNA expression in whole blood of Chinese healthy individuals. P-values are calculated based on Kruskal-Wallis non-parametric test.

We also evaluated the predicted effect of the candidate SNPs on eQTL in cells from whole blood, using an unpublished Asian population dataset and uncovered significant effects on *TNFSF15* expression (Table S6), which were specific to this gene, with no eQTL effects predicted on other genes nearby, including *TNFSF8* (data not shown). The SNP rs6478109 was significantly associated to the expression levels of *TNFSF15* in independent datasets as determined in whole blood (P = 5.41 × 10^−13^, Fig. 4), and isolated monocytes. The A allele of rs6478109, which was associated with risk for CD, and protection for leprosy, associated strongly with increased levels of *TNFSF15*. Additionally, rs4979462 also showed an eQTL effect on *TNFSF15* in whole blood (P = 1.19 × 10^−4^), however this effect was abolished when conditioning on the effect of rs6478109 (P_conditional_ = 0.692), while the eQTL effect of rs6478109 remained strong after conditioning on rs4979462 (P_conditional_ = 1.11 × 10^−9^, Table S6). Together with the results from the analysis of transcriptional regulatory activity, these data indicate that rs6478109 genotype has significant impact on *TNFSF15* expression in immune cells.

## Discussion

Several GWAS studies have hinted at associations between the 9q32 region, and specifically the *TNFSF15* locus, and diverse pathological states including inflammatory diseases of the gut and infectious diseases such as leprosy. While these studies are useful for highlighting genomic regions of interest, their necessarily-broad view means they have insufficient resolution and stringency to definitively identify likely causative variants. Fine-mapping approaches enable us to examine a small region of the genome in greater detail, and combined with advanced imputation methods, stringent quality control, and functional annotation/experimentation have proven capable of linking SNPs with pathological mechanisms involved in disease. Here we performed a fine-mapping study of the *TNFSF15* locus in three large disease samples of leprosy (9,619 cases and 12,896 controls of Chinese ethnicity), CD (1,576 cases and 3,013 controls of Korean ethnicity) and PBC (1,594 cases and 1,529 controls of Japanese ethnicity); through conditional and haplotype analyses, we clearly demonstrated that there are two independent associations at both rs6478108/rs6478109 and rs4979462 for risk of CD and leprosy, and a single association at rs4979462 for risk of PBC. While an association between risk of PBC and variance at rs6478108 was observed, this effect was purely due to its LD with rs4979462 and was accordingly abolished by conditioning on rs4979462. Thus rs4979462 plays a role in all three diseases, while variance at rs6478108/rs6478109 has a genetic risk effect in CD and leprosy, but not PBC.

Interestingly the disease associations with specific SNPs in the *TNFSF15* locus show opposite effects in leprosy and CD/PBC: the T allele at rs4979462 significantly increases risk of CD and PBC, but significantly decreases risk of leprosy; while at rs6478108, which only showed independent associations with CD and leprosy, the T allele significantly increases risk of CD, but significantly decreases risk of leprosy. A similar phenomenon has been reported in studies of CD risk and norovirus susceptibility, where individuals that carry two copies of the CD risk allele at rs601338 in *FUT2* are protected from norovirus infection^12^,^25^: and in celiac disease, the positively selected risk variant in *SH2B3* might also play a protective role against bacterial infections^26^. Together with our findings, these observations seem to align with the “hygiene hypothesis”, which postulates that alleles whose main role was to maintain protective immune responses against infection in the past (when pathogen exposure was high), have now become deleterious in the modern “hygienic” lifestyle, where pathogen exposure is relatively low, instead contributing to autoimmune and inflammatory disease development^27^,^28^,^29^. Recently, a study identified 21 loci for inflammatory-disease susceptibility that exhibit signatures of recent positive selection^27^, further supporting the “hygiene hypothesis”. However, *TNFSF15* was not among these 21 loci, and we could not find any evidence of recent selection in the current study. A more thorough analysis for selection signatures is required to understand the evolutionary forces that might have shaped the variance within this locus.

In order to evaluate the likelihood that rs6478108 and rs4979462 were directly linked with disease causation, we estimated their potential for regulatory effects upon *TNFSF15.* By intersecting rs6478108 and rs4979462, as well as those SNPs within the *TNFSF15* locus in high LD with rs6478108 and rs4979462, with the regulatory information from the ENCODE and ROADMAP Projects, we found that both rs4979462 and rs6478109 (which is in perfect LD with rs6478108) are much more likely to exhibit a regulatory effect on gene expression than other variants within the region. While both SNPs are predicted to exhibit regulatory function in gastrointestinal tissues, rs6478109-related regulatory sequence was likely to be functional in immune cells from blood, whereas rs4979462-related regulatory sequence was likely to be functional in epithelial and connective tissues. The cell and tissue specific regulatory roles of rs6478109 and rs4979462 provide biological insight into the differential associations of these two SNPs with three different diseases: because the regulatory sequences of both SNPs are functional in gastrointestinal cells, we would anticipate that both SNPs would exhibit a risk effect in CD; while the role of rs6478109-related regulatory sequence in immune cells makes sense of the SNP’s association with leprosy, where immunity to the pathogenic agent will play a major role. That rs4979462 is also associated with risk of leprosy suggests that the transcriptional regulation of *TNFSF15* in gastrointestinal or epithelial/connective tissues might play a previously-unappreciated role in the development of leprosy. Moreover, the positive association of rs4979462, and the negative association of rs6478108, with risk of PBC, suggest that TNFSF15 may influence the development of PBC predominantly through its function in epithelial and connective tissues.

We also assessed the likely effects of rs6478108 and rs4979462 on eQTLs and found that rs6478109 had a significant and specific eQTL effect on *TNFSF15* expression in whole blood and in isolated monocytes. This indicates that *TNFSF15*, and not other genes within the 9q32 locus, such as *TNFSF8*, is likely to be the causative pathogenic disease gene of the locus. Interestingly, the A allele of rs6478109 was associated with higher expression of *TNFSF15* in blood; given that the same allele was associated with increased risk of CD, and decreased risk of leprosy, it follows that high expression of *TNFSF15* will increase CD risk, but decrease leprosy risk. Although there was no evidence for an eQTL effect of rs4979462 here, we previously showed that increased expression of *TNFSF15* may be driven by the PBC-susceptible allele of rs4979462, as a result of the generation of a novel NF-1 binding site by the A allele in human T-cell Jurkat and bile duct HuCCT1 cell lines^30^. However, the functional role of rs4979462 in gastrointestinal, epithelial and connective tissues remain to be elucidated.

In summary, we have performed a fine-mapping and functional annotation analysis of the *TNFSF15* locus in leprosy, CD and PBC. Two independent associations have been discovered within the locus, with complex patterns of genetic effects across the three diseases: one variant shows pleiotropic effect across the three diseases, and the other plays a role in CD and Leprosy but not PBC. For both associations, the genetic effect is opposite in the autoimmune/inflammatory diseases and infection. Further regulatory function annotation and eQTL analysis both confirmed *TNFSF15* as the most likely pathogenic gene of the locus, and revealed strong evidence for the regulatory functionality of both associations in a cell/tissue-specific fashion. Our study highlights the potentially diverse function of TNFSF15 in immune regulation, gastrointestinal cells and epithelial/connective tissues, and indicates that increased expression of *TNFSF15* may simultaneously confer protection against infections, such as leprosy, while increasing the risk for immune and inflammatory diseases.

## Methods

### Study subjects

#### Chinese leprosy

The Chinese leprosy GWAS dataset consisted of 1,548 leprosy cases and 2,150 healthy controls that were derived from 2 previously published studies^14^,^15^ (Supplementary Methods). As for the initial replication analysis, 6 SNPs (rs6478108, rs6478109, rs4263839, rs7848647, rs4366152 and rs4979462) were genotyped in an additional 2,812 leprosy cases and 5,464 controls, consisting of 2,701 cases and 4,602 controls of Northern Chinese Han and 111 cases and 862 controls of Southern Chinese Han. As for the second replication analysis, two SNPs (rs6478108 and rs4979462) were genotyped in the second independent sample of 5,259 leprosy cases and 5,282 controls, consisting of 444 cases and 2,418 controls of northern Chinese Han and 4,815 cases and 2,864 controls of southern Chinese Han. The study was approved by the institutional review board of the Shandong Provincial Institute of Dermatology and Venereology, with informed consent obtained from all individuals.

#### Korean CD

The Korean CD dataset was derived from 2 previously published studies^10^,^11^: 532 cases and 739 controls genotyped on a GWAS chip (Illumina OmniExpress chip), and 729 cases and 469 controls genotyped on Illumina Immunochip. 385 cases and 305 controls overlapped between the two studies, which means information on GWAS as well as immune-related SNPs are available for these samples; we merged these markers before imputation. As for samples that were not typed on both GWAS and Immunochip, we imputed them separately. Subsequently, an independent set of 722 cases and 2124 controls were used for the replication analysis. The study was approved by the institutional review board of the Asan Medical Center, with informed consent obtained from all individuals.

#### Japanese PBC

The discovery dataset of Japanese PBC included 509 cases and 493 controls: an additional 313 cases and 437 controls were involved in the first replication study, with a further 772 cases and 599 controls involved in the second replication study. These sets were derived from our previously-published GWAS^9^. The study was approved by the research ethics committee of the Graduate School of Medicine, The University of Tokyo, with informed consent obtained from all individuals.

The collection of blood samples and clinical information from all subjects was undertaken in accordance with the Declaration of Helsinki.

### Phasing and Imputation of the GWAS datasets

Imputation analysis of the *TNFSF15* locus was performed in each of the five previously-published GWAS datasets separately: two leprosy datasets from Chinese ethnicity individuals^14^,^15^, two CD datasets from Korean ethnicity individuals^10^,^11^ and one PBC dataset from Japanese ethnicity individuals^9^. The analysis followed a two-level imputation protocol, integrated by reference panels from the 1000 Genomes Project Phase I integrated variant set v3 (March 2012 release, Level 1) and population-specific sequenced data of each population (Level 2). Technical details of phasing, imputation and reference datasets can be found in Supplementary Methods. For each population dataset, the phased and imputed region encompassed chromosome 9 from positions 117.25 to 117.72 Mb (coordinates based on NCBI Build 37). After imputation and QC, there were 1,928 SNPs and 34 indels in the Chinese leprosy dataset, 1,314 SNPs and 16 indels in the Korean CD dataset and 1,463 SNPs and 179 indels in the Japanese PBC dataset.

### Genotyping in replication datasets

SNP validation was performed by genotyping using Sequenom MassArray platform or running TaqMan assays; details in Supplementary Methods.

### Whole blood eQTL dataset

We associated gene expression data obtained from whole blood mRNA from 206 Chinese ethnicity volunteers in the context of their genotypes for the SNPs under investigation in this report. (*Unpublished dataset*). RNA was isolated from blood collected in Tempus tubes (Life Technologies, Carlsbad, Calif) and gene expression was estimated by analysing on the Illumina HumanHT-12-v4 Expression Bead Chip (Illumina, San Diego, Calif). The genotypes were determined using the Illumina Human Omni5Quad chip. Only Illumina probes free of any SNPs were used to determine the expression level of the genes to avoid allele-specific artefacts. Probes used for analysis included the following: ILMN_1759501 (*TNFSF15*), ILMN_1761778 (*TNFSF8*).

### Statistical Analysis

#### Association analysis

Association analysis was performed in each dataset in each diseased population. Genotyped SNPs were converted to genotype dosages to be analysed together with the dosages of imputed SNPs. The logistic regression under the additive model as implemented in SNPTEST^31^ version 2.4.1 was performed. For the analysis of individual datasets please see Supplementary Methods. For the joint analysis of the combined discovery and validation samples, fixed-effects meta-analysis was used to combine the statistical results from each dataset within each diseased population, performed in either META^32^ version 1.3.2 or Metasoft^33^ version 2.0.0.

#### Haplotype analysis

For the haplotype-based association analysis, we first phased the haplotypes using PHASE^34^,^35^ version 2.1.1 program, before performing the association in R under a logistic regression model, treating one haplotype as a reference.

#### Power calculation

To calculate the power of the studies to detect association in each haplotype, we used Quanto version 1.2.4^36^.

#### e-QTL analysis

Associations between SNP genotypes and gene expression were evaluated using Kruskal-Wallis test on geometric mean intensity values implemented in R kruX package^37^.

### Functional annotations

We first determined the critical region of the *TNFSF15* locus that contained the SNPs and indels in high linkage disequilibrium (LD, r^2^≥0.8) with rs6478108 and rs4979462 using the LD information provided by HaploReg v2^38^ on the 1000 Genomes Phase 1 Asian (ASN) population, as well as the LD values calculated within each of the three population GWAS datasets. The critical region encompassed chr9: 117535517-117599093, containing 18 noncoding SNPs (no indels) in high LD with rs6478108 and rs4979462. The initial functional annotations of non-coding SNPs were carried out by asking whether a SNP causes splicing mis-regulation and so alternative *TNFSF15* transcript abundance, using the SPANR web-based tool^39^. We then asked whether the SNP overlapped with any deoxyribonuclease (DNase) peaks using data from 131 different tissues/cells present in the ENCODE and ROADMAP databases. The DNase analysis identified 81 tissues/cells where positive DNase peaks (>30) overlapped with any of the 18 SNPs. For each of the 81 tissues/cells, we further functionally annotated the SNPs using WashU EpiGenome Browser^40^ for visualization and manual inspection of overlapping histone modifications, RNA sequencing (RNA-Seq) and transcription factor ChIP-Seq peaks (where available) against positive DNase peaks. We also included the functionality scores (based on the integration of multiple high throughput datasets for DNA features and regulatory elements) and the position-weight matrix for transcription factor binding from the RegulomeDB^41^ into the functional annotation.

We also evaluated the cis-eQTL effect of the 18 SNPs (P < 0.01) on genes located within 1 Mb of these SNPs using data from multiple cell types, including B-cells, monocytes, T-effector, T-regulator cells^42^ (15 samples each), neutrophils^43^ (96 samples), and whole blood (206 samples, unpublished), using the dataset obtained from a Singaporean Chinese population. Finally, published literature on functional analysis^26^,^47^ of these SNPs in any specific cells and disease context were also evaluated and summarized^44^,^45^,^46^.

## Additional Information

**How to cite this article**: Sun, Y. *et al.* Fine-mapping analysis revealed complex pleiotropic effect and tissue-specific regulatory mechanism of TNFSF15 in primary biliary cholangitis, Crohn’s disease and leprosy. *Sci. Rep.* **6**, 31429; doi: 10.1038/srep31429 (2016).

## Supplementary Material

Supplementary Information

Supplementary Table S5

## Figures and Tables

**Table 1:** Single-variant association results (dosage model) from the imputed GWAS datasets of three diseases.

SNP			Chinese Leprosy (1548 cases, 2150 controls)				Korean CD (854 cases, 889 controls)				Japanese PBC (1594 cases, 1529 controls)			
Population	RSID	A1/A2	F_A	F_U	P	OR	F_A	F_U	P	OR	F_A	F_U	P	OR
CHN	rs4366152	C/T	0.435	0.519	2.39E-14	0.66	0.713	0.521	6.61E-28	2.25	0.70	0.61	1.22E-12	1.48
KOR	rs6478108	T/C	0.427	0.510	6.47E-14	0.67	0.711	0.515	3.77E-29	2.29	0.70	0.62	3.22E-11	1.45
JPN	rs4979462	T/C	0.209	0.271	6.39E-10	0.68	0.525	0.353	7.58E-23	2.02	0.57	0.46	1.37E-16	1.54

CHN, Chinese; KOR, Korean; JPN, Japanese; A1, allele 1; A2, allele 2; F_A, frequency of A1 in the cases; F_U, frequency of A1 in the controls; P, P-value from fixed effects meta-analysis of studies within each diseased population; OR, odds ratio per copy of A1.
