# Supplementary material for: Fine-mapping analysis revealed complex pleiotropic effect and tissue-specific regulatory mechanism of TNFSF15 in primary biliary cholangitis, Crohn’s disease and leprosy
Source: Sci Rep. 2016 Aug 10;6:31429. doi: 10.1038/srep31429 (PMC4979016; doi:10.1038/srep31429)

Supplementary Materials

Fine-mapping analysis revealed complex pleiotropic effect and tissue-specific regulatory mechanism of TNFSF15 in primary biliary cholangitis, Crohn's disease and leprosy

Author list:

Yonghu Sun[1,2,3,14], Astrid Irwanto[4,14], Licht Toyo-oka[5], Myunghee Hong[6], Hong Liu[1,2,3], Anand Kumar Andiappan[7], Hyunchul Choi[6], Yuki Hitomi[5], Gongqi Yu[1,2,3], Yongxiang Yu[1,2,3], Fangfang Bao[1,2,3], Chuan Wang[1,2,3], Xian Fu[1,2,3], Zhenhua Yue[1,2,3], Honglei Wang[1,2,3],Huimin Zhang[1,2,3], Minae Kawashima[5], Kaname Kojima[8], Masao Nagasaki[8], Minoru Nakamura[9], Suk-Kyun Yang[10], Byong Duk Ye[10], Yosua Denise[4,11], Olaf Rotzschke[7], Kyuyoung Song[6,15], Katsushi Tokunaga[5,15], Furen Zhang[1,2,3,12,13,15], Jianjun Liu[4,15]

Affliations:

[1]Shandong Provincial Institute of Dermatology and Venereology, Shandong Academy of Medical Sciences, Jinan, Shandong 250000, China

[2]Shandong Provincial Key Laboratory for Dermatovenereology, Jinan, Shandong 250000, China

[3]Shandong Provincial Hospital for Skin Diseases, Shandong University, Jinan, Shandong 250000, China

[4]Human Genetics, Genome Institute of Singapore, Singapore 138672, Singapore

[5]Human Genetics, University of Tokyo, Graduate School of Medicine, Tokyo 113-0033, Japan

[6]Biochemistry and Molecular Biology, University of Ulsan College of Medicine, Seoul 138736, Korea [7]Immune Regulation, Singapore Immunology Network, Singapore 138648, Singapore

[8]Integrative Genomics, Tohoku Medical Megabank Organization, Tohoku University, Sendai, 980-8573, Japan

[9]Clinical Research Center, National Hospital Organization Nagasaki Medical Center, Onuma, Nagasaki 856-8562, Japan

[10]Gastroenterology, Asan Medical Center, University of Ulsan College of Medicine, Seoul 138736, Korea

[11]Faculty of Biotechnology, Atma Jaya Catholic University of Indonesia, South Jakarta 12930, Indonesia

[12]School of Medicine, Shandong University, Jinan, Shandong 250000, China.

[13]National Clinical Key Project of Dermatology and Venereology, Jinan, Shandong 250000, China.

[14]These authors contributed equally to this work

[15]These authors equally directed this work

Corresponding author: Jianjun Liu

Address: 60 Biopolis Street, Genome, #02-01, Singapore 138672

Email: liuj3@gis.a-star.edu.sg

Tel/Fax: +65-68088088/+65-68088304

Supplementary Methods

Chinese leprosy study subjects.

Data included in this study consisted of two published studies. The first study had 706 subjects with leprosy and 1,225 healthy controls of northern Chinese Han descent [1,2], while the second one consisted of 842 subjects with leprosy and 925 healthy controls of Chinese population (including minority groups) [1,2].

Phasing and Imputation of the GWAS datasets

Phasing was performed using SHAPEIT2 version 2 [3] software, which was followed by a two-level imputation using IMPUTE [4] version 2.2.2 and two reference panels from the 1000 Genomes Project Phase I integrated variant set v3 (March 2012 release, Level 1) and population-specific sequenced data of each population (see below, Level 2).

Sequencing data of Chinese population was obtained from a targeted sequencing analysis of 168 individuals (89 leprosy cases and 79 controls) from Northern Chinese Han population (unpublished data) using Illumina Miseq platform. The average coverage was 39x. GATK was used to call SNPs, perform local realignment to identify insertions-deletions (indels) and remove clusters of false positive SNPs on the basis of discrepancy between forward and reverse strand reads. The Korean sequenced data of 392 individuals was obtained from the Korean Reference Genome Database (see Web Resources) at Korea National Institute of Health. In brief, the whole genome sequencing of these Korean samples was done using Illumina's HiSeq 2000 at an average read depth of 30x. The reference panel for Japanese population, 1KJPN [5], was built

based on the sequencing data for 1,070 Japanese healthy individuals from HiSeq 2500 with PCR-free protocol with the average read coverage of 32.4x, and variants in the panel were called with Bcftools [6] version 0.1.17-dev after the alignment of the sequence data to GRCh37/hg19 with the decoy sequence (hs37d5) with Bowtie [7] version 2.1.0. The called variants that deviated from Hardy-Weinberg equilibrium in controls at $P < 1\times10^{-4}$ were filtered out, and the remaining variants were phased with SHAPEIT2 version 2 [3].

The imputed genotypes were filtered by removing monomorphic SNPs, SNPs with info score <0.5, and SNPs that deviated from Hardy-Weinberg equilibrium in controls at $P < 1\times10^{-5}$.

Genotyping in replication datasets

Chinese leprosy. Six SNPs were genotyped at the Shandong Provincial Key Laboratory for Dermatovenereology by using Sequenom MassARRAY platform according to manufacturer's instructions. Conducted in the same laboratory, the second replication phase included two SNPs using TaqMan genotyping platform on a 7900 HT Fast Real-Time PCR System (Applied Biosystems) according to manufacturer's instructions.

Korean CD. Validation of rs6478108 and rs4979462 were done using the TaqMan SNP genotyping assays.

Japanese PBC. Four SNPs were genotyped by using TaqMan genotyping platform on a Light-Cycler (Roche) according to manufacturer's instructions.

Statistical Analysis

Association analysis. For the analysis of the Chinese leprosy GWAS datasets, we included the

first five principal components as covariates to control for the impact of population stratification as described before[2]. Subsequently, follow-up samples that were used for validation were also separated into northern and southern Chinese before performing association analysis.

As for the analysis of the Korean CD and Japanese PBC datasets, the cases and controls were genetically matched [8-11], and no PCs were included as covariates in association analysis.

References

1. Zhang, F.-R. *et al.* Genomewide association study of leprosy. *N. Engl. J. Med.* **361,** 2609–2618 (2009).
2. Liu, H. *et al.* Discovery of six new susceptibility loci and analysis of pleiotropic effects in leprosy. *Nat Genet* **47,** 267–271 (2015).
3. Delaneau, O., Zagury, J.-F. & Marchini, J. Improved whole-chromosome phasing for disease and population genetic studies. *Nature Methods* **10,** 5–6 (2013).
4. Howie, B. N., Donnelly, P. & Marchini, J. A flexible and accurate genotype imputation method for the next generation of genome-wide association studies. *PLoS Genet* **5,** e1000529 (2009).
5. Nagasaki, M. *et al.* Rare variant discovery by deep whole-genome sequencing of 1,070 Japanese individuals. *Nat Comms* **6,** 8018 (2015).
6. Li, H. *et al.* The Sequence Alignment/Map format and SAMtools. *Bioinformatics* **25,** 2078–2079 (2009).
7. Langmead, B., Trapnell, C., Pop, M. & Salzberg, S. L. Ultrafast and memory-efficient alignment of short DNA sequences to the human genome. *Genome Biol.* **10,** R25 (2009).
8. Nakamura, M. *et al.* Genome-wide association study identifies TNFSF15 and POU2AF1 as susceptibility loci for primary biliary cirrhosis in the Japanese population. *Am. J. Hum. Genet.* **91,** 721–728 (2012).
9. Nakamura, M. *et al.* Genome-wide Association Study Identifies TNFSF15 and POU2AF1 as Susceptibility Loci for Primary Biliary Cirrhosis in the Japanese Population. *The American Journal of Human Genetics* **91,** 721–728 (2012).
10. Yang, S. K. *et al.* Genome-wide association study of Crohn's disease in Koreans revealed three new susceptibility loci and common attributes of genetic susceptibility across ethnic populations. *Gut* **63,** 80–87 (2013).
11. Yang, S.-K. *et al.* Immunochip Analysis Identification of 6 Additional Susceptibility Loci for Crohn's Disease in Koreans. *Inflammatory Bowel Diseases* **21,** 1–7 (2015).

Supplementary Tables

Supplementary Table 1 Haplotype analysis of rs6478108-rs4979462 in GWAS datasets

Haplo-Type*	Chinese Leprosy (1548 cases/2150 controls)		Korean CD (854 cases/889 controls)		Japanese PBC (822 cases/930 controls)	
	P	OR	P	OR	P	OR
TC	1.63E-06	0.75	2.01E-09	1.90	0.05	1.25
TT	3.56E-11	0.68	1.73E-30	2.57	2.94E-12	1.68

*CC haplotype is set as reference haplotype.

Supplementary Table 2 Follow-up of top GWAS variants in an independent Chinese leprosy dataset

SNP				GWAS (1548 cases /2150 controls)					Replication stage 1 (2812cases/5464controls)					Meta	
SNP	Pos (hg19)	Minor	Major	F_A	F_U	P	OR	SE	F_A	F_U	P	OR	SE	P	OR
rs6478108	117558703	C	T	0.427	0.510	6.47E-14	0.67	0.05	0.44	0.50	1.50E-12	0.79	0.03	2.73E-26	0.74
rs6478109	117568766	G	A	0.431	0.515	5.99E-14	0.67	0.05	0.45	0.50	2.60E-12	0.79	0.03	7.60E-26	0.74
rs4263839	117566440	G	A	0.435	0.519	2.48E-14	0.66	0.05	0.45	0.51	3.13E-12	0.80	0.03	3.75E-26	0.74
rs7848647	117569046	C	T	0.431	0.515	7.20E-14	0.67	0.05	0.45	0.51	3.27E-12	0.80	0.03	3.39E-17	0.76
rs4366152	117564875	C	T	0.435	0.519	2.39E-14	0.66	0.05	0.45	0.51	5.76E-12	0.80	0.03	7.95E-26	0.74
rs4979462	117567013	T	C	0.208	0.271	6.39E-10	0.68	0.06	0.23	0.26	1.54E-05	0.85	0.04	1.13E-25	0.74

Supplementary Table 3 Power calculation for haplotype analysis (CC as reference haplotype) assuming OR of 1.5

Alpha	Power for TC haplotype			Power for TT haplotype		
	Chinese Leprosy	Korean CD	Japanese PBC	Chinese Leprosy	Korean CD	Japanese PBC
0.1	1	1	0.9994	1	1	1
0.05	1	1	0.9984	1	1	1
0.01	1	0.9999	0.9901	1	1	1
0.001	1	0.9987	0.9471	1	1	1
0.0001	1	0.9921	0.8455	1	1	0.9997
0.00001	1	0.9704	0.6881	1	1	0.9984
5.00E-08	1	0.8031	0.2934	1	0.9979	0.9725

Supplementary Table 4 SNPs in LD with main association and their annotations

No	SNP	Main association (Proxy)	r^2 to proxy	D' to proxy	Position (hg19)	dbSNP function annotation	Affect splicing*	Overlap DNase peak in any of the 131 cell/tissues
1	rs4979462	rs4979462	1	1	117567013	intronic	no	yes
2	rs55951892	rs4979462	1	1	117575913	7.5kb from 5' of TNFSF15	no	no
3	rs56006049	rs4979462	0.99	1	117579050	11kb 5' of TNFSF15	no	no
4	rs1322057	rs4979462	1	1	117578374	10kb 5' of TNFSF15	no	no
5	rs56211063	rs4979462	0.99	1	117585897	17kb 5' of TNFSF15	no	yes
6	rs55768522	rs4979462	1	1	117574220	5.8kb 5' of TNFSF15	no	no
7	rs55717217	rs4979462	0.89	0.96	117554069	intronic	no	no
8	rs55682128	rs4979462	0.89	0.96	117537523	14kb 3' of TNFSF15	no	no
9	rs911604	rs4979462	0.83	0.96	117589257	21kb 5' of TNFSF15	no	no
10	rs78898421	rs4979462	0.83	0.92	117594267	26kb 5' of TNFSF15	no	no
11	rs6478108	rs6478108	1	1	117558703	intronic	no	no
12	rs4366152	rs6478108	1	1	117564875	intronic	no	no
13	rs10817678	rs6478108	1	1	117579457	11kb 5' of TNFSF15	no	no
14	rs4263839	rs6478108	0.97	1	117566440	intronic	no	yes
15	rs6478109	rs6478108	0.98	1	117568766	359bp 5' of TNFSF15	no	yes
16	rs7848647	rs6478108	0.98	1	117569046	639bp 5' of TNFSF15	no	yes
17	rs7869487	rs6478108	0.87	1	117580914	13kb 5' of TNFSF15	no	no
18	rs4372078	rs6478108	0.84	1	117563687	intronic	no	no

r^2 and D’ displayed are calculated based on 1000 Genomes Phase 1 ASN population.

* Results obtained from SPANR web-based tool (Xiong HY, Alipanahi B, Lee LJ, Bretschneider H, Merico D, Yuen RKC, et al. (2015). The human splicing code reveals new insights into the genetic determinants of disease. *Science* 347: 1254806–6).

Supplementary Table 5 Functional annotations of candidate causal SNPs.
In a separate spreadsheet.

Supplementary Table 6 Expression-quantitative trait loci for *TNFSF15*

Blood cell types	SNP and P-value	
	rs6478109	rs4979462
**Unconditional**		
Whole Blood (N=206)	5.41×10^{-13}	1.19×10^{-4}
Monocytes (N=15)	4.00×10^{-3}	0.912
B-cells (N=15)	0.637	0.129
Neutrophils (N=97)	0.619	0.470
T-effector cells (N=15)	0.848	0.789
T-regulator cells (N=15)	0.586	0.174
**Conditional on rs6478109**		
Whole Blood (N=206)	NA	0.692
Monocytes (N=15)	NA	0.348
**Conditional on rs4979462**		
Whole Blood (N=206)	1.11×10^{-9}	NA
Monocytes (N=15)	3.55×10^{-3}	NA

SUPPLEMENTARY FIGURES

Supplementary Figure 1 GWAS regional association plots of TNFSF15

a) Chinese Leprosy (1548 cases and 2150 controls)

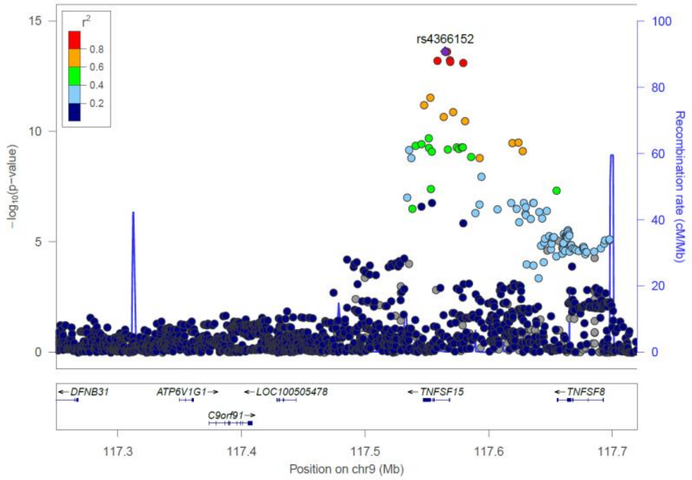

b) Korean CD (854 cases and 889 controls)

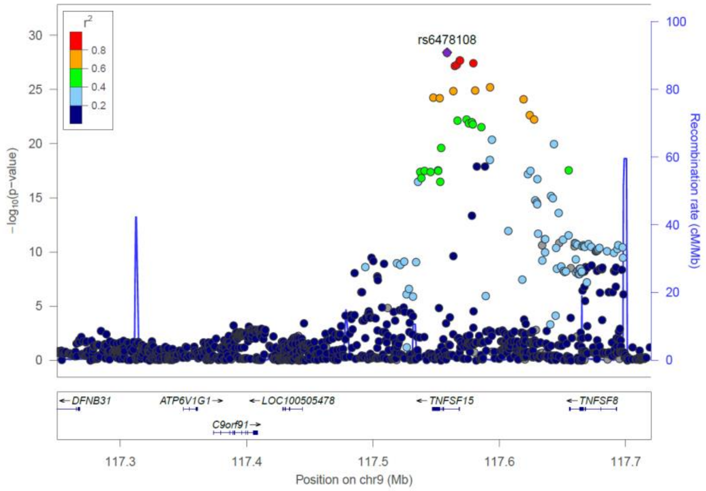

c) Japanese PBC (1594 cases and 1529 controls)

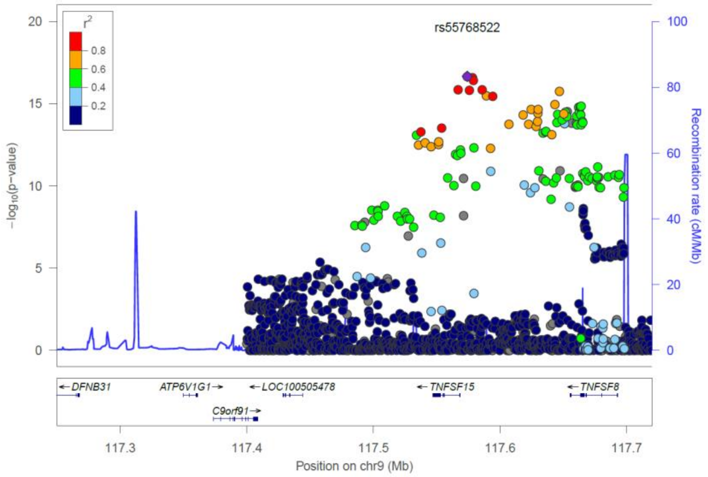

Supplementary Figure 2 Linkage disequilibrium of top SNPs in three populations

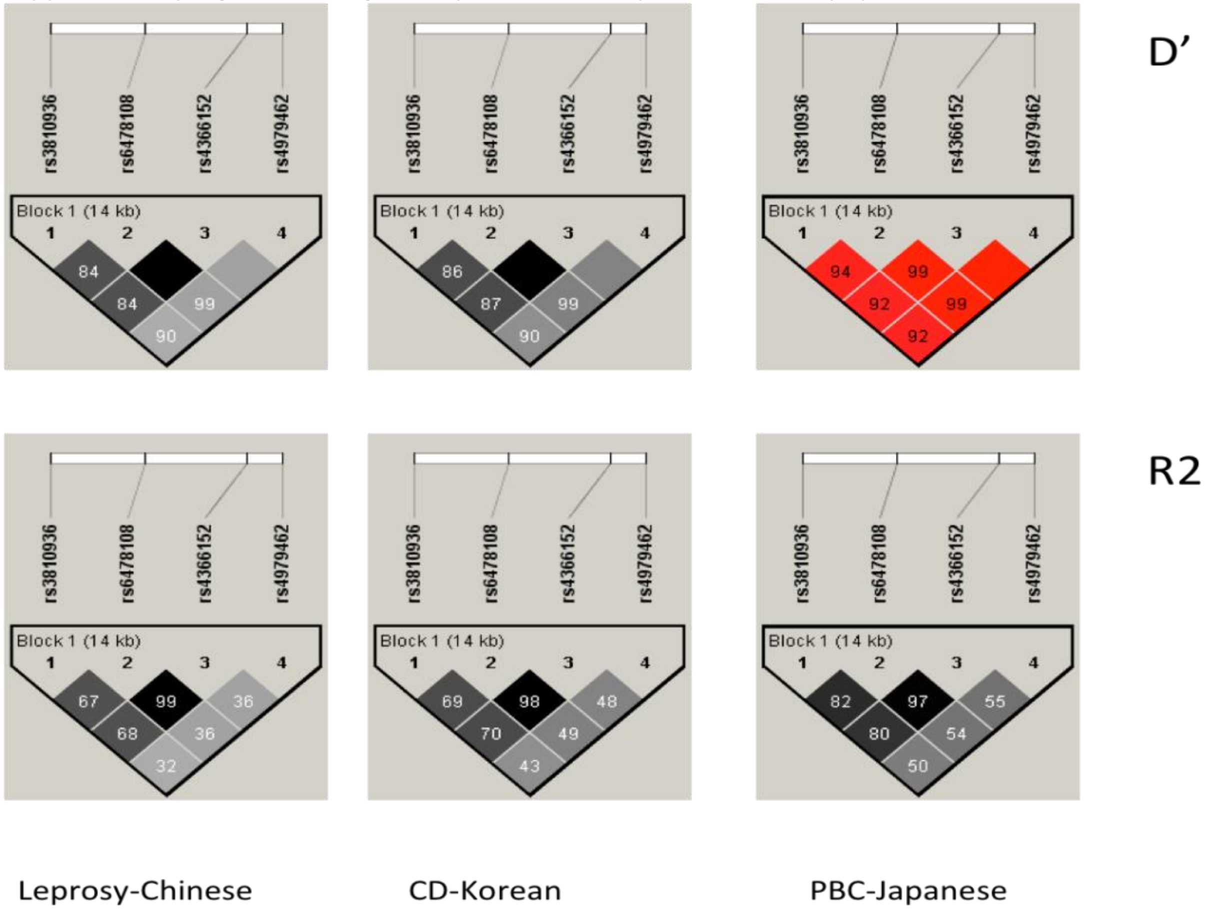

Supplementary Figure 3 Conditional Analysis of TNFSF15 in Korean CD and Chinese Leprosy

Korean CD
854 cases and 889 controls

Chinese Leprosy
1548 cases and 2150 controls

Unconditional

A

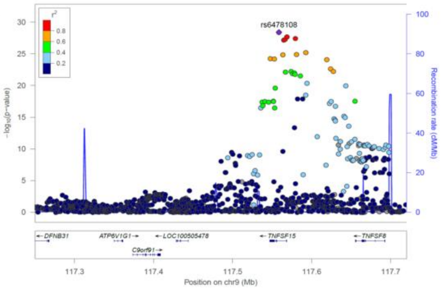

B

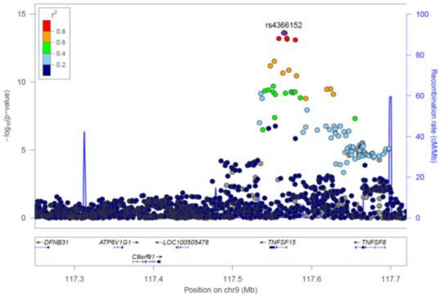

Condition on rs6478108

C

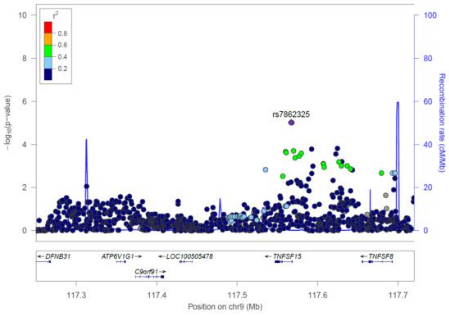

D

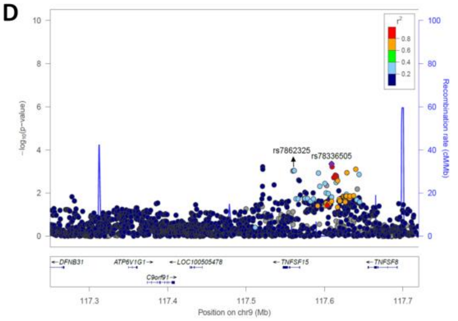

Condition on rs4979462

E

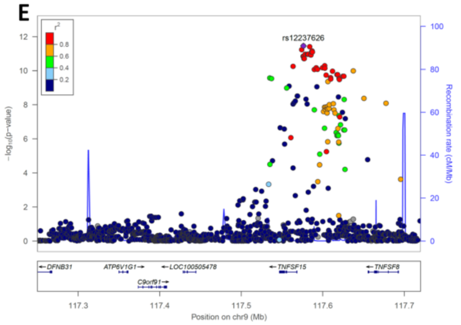

F

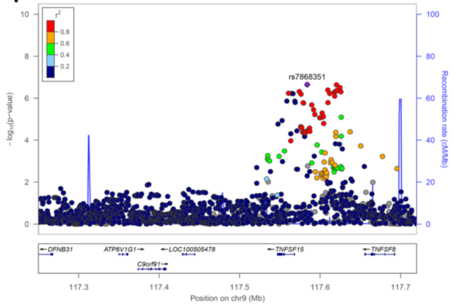

Condition on rs6478108 and rs7862325

G

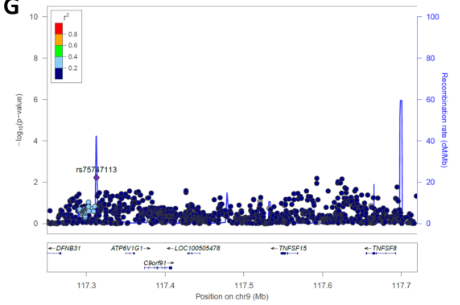

H

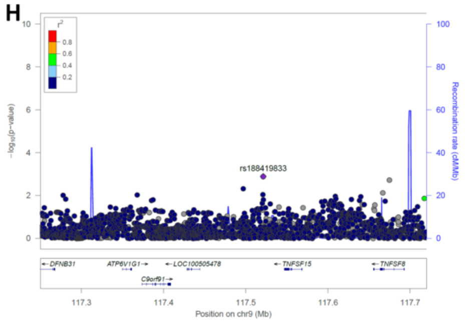

Supplementary Figure 4 Conditional Analysis of TNFSF15 in Japanese PBC (1594 cases and 1529 controls)

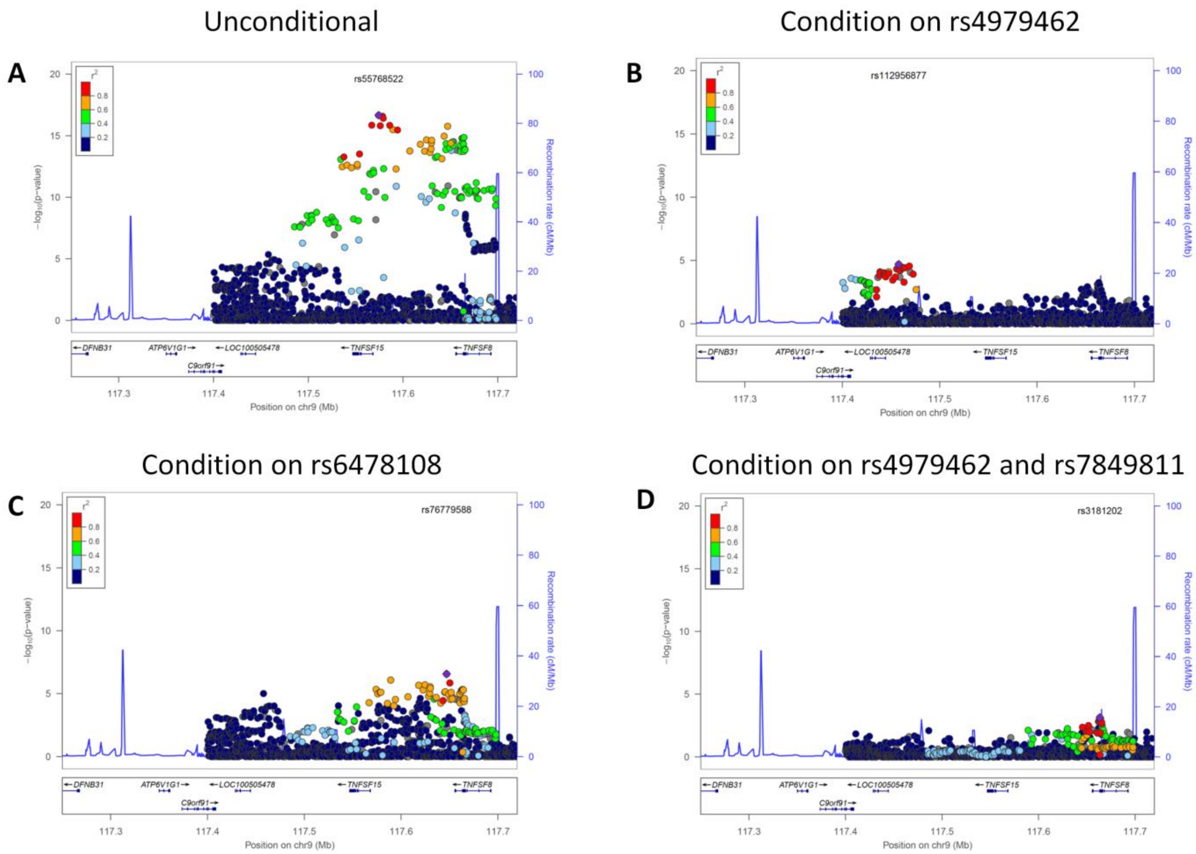

Notes: rs55768522 is in perfect linkage disequilibrium with rs4979462 (r2=1).

Supplementary Figure 5 DNase peaks in 131 tissues/cells from ENCODE and Roadmap within TNFSF15 locus.

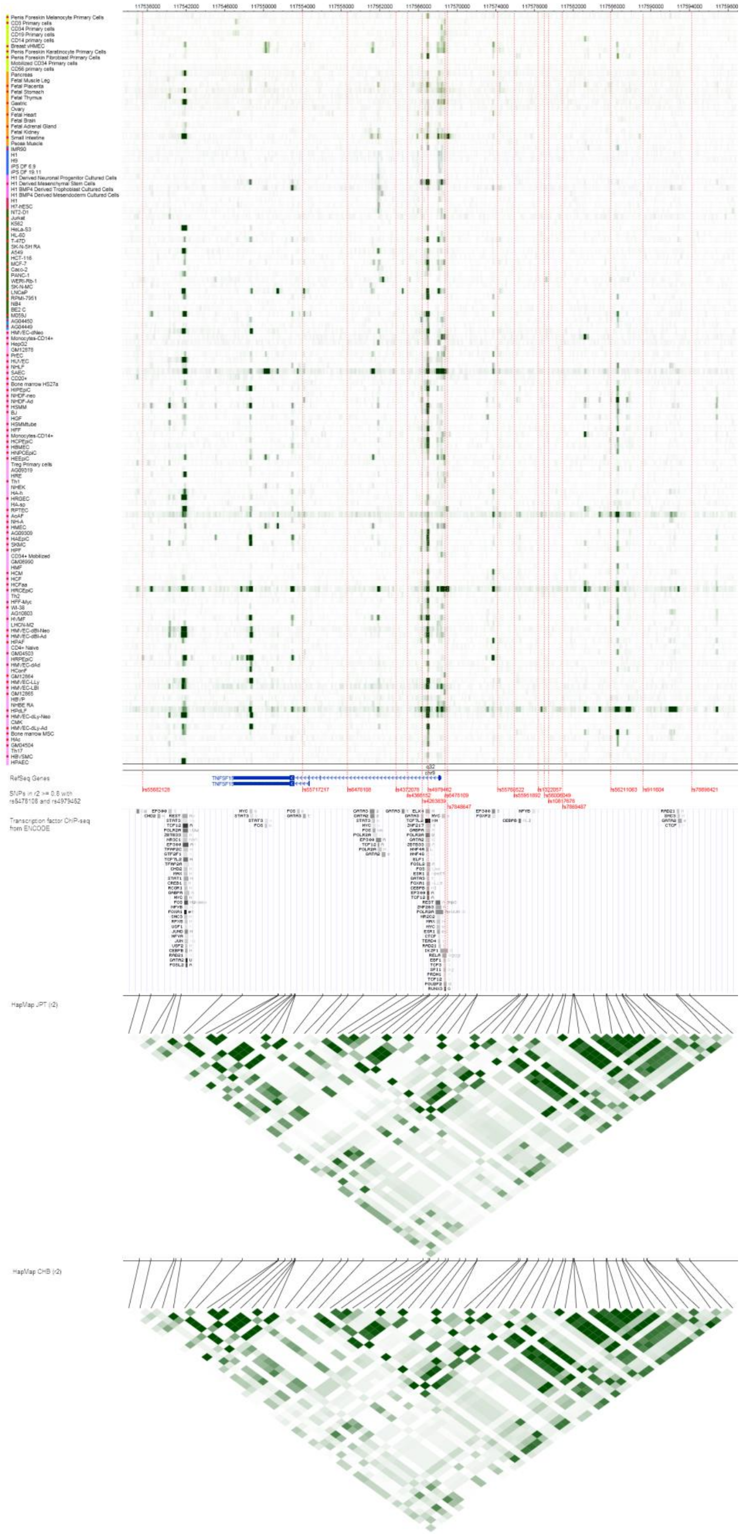

Note: Red dots on the left side of the panel indicate cells with positive DNase peaks overlapping SNPs

Supplementary Figure 6 Roadmap and ENCODE Annotation in Stromal and Connective Tissues

a) Penis foreskin

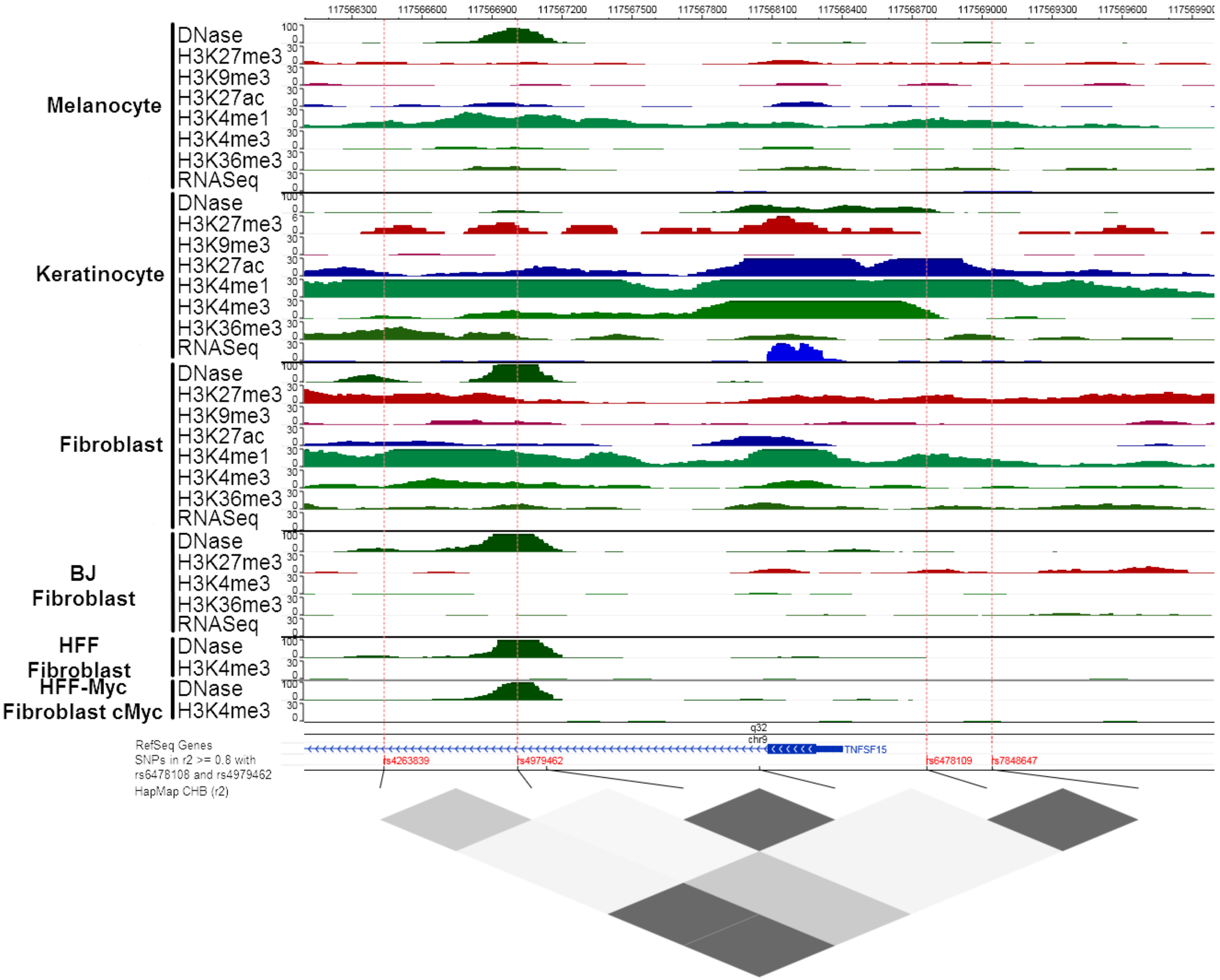

b) Lung fibroblast

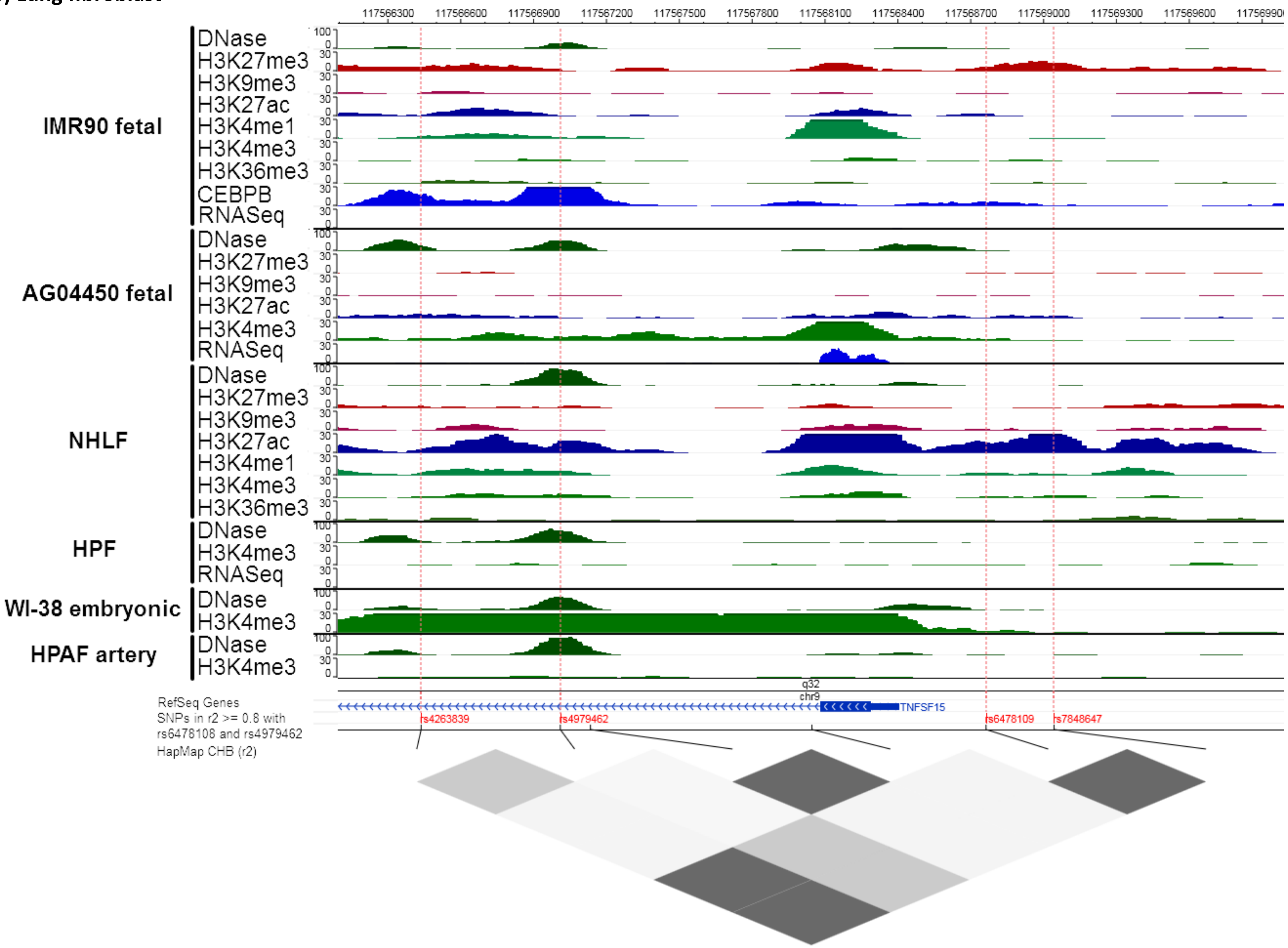

c) Skin fibroblast

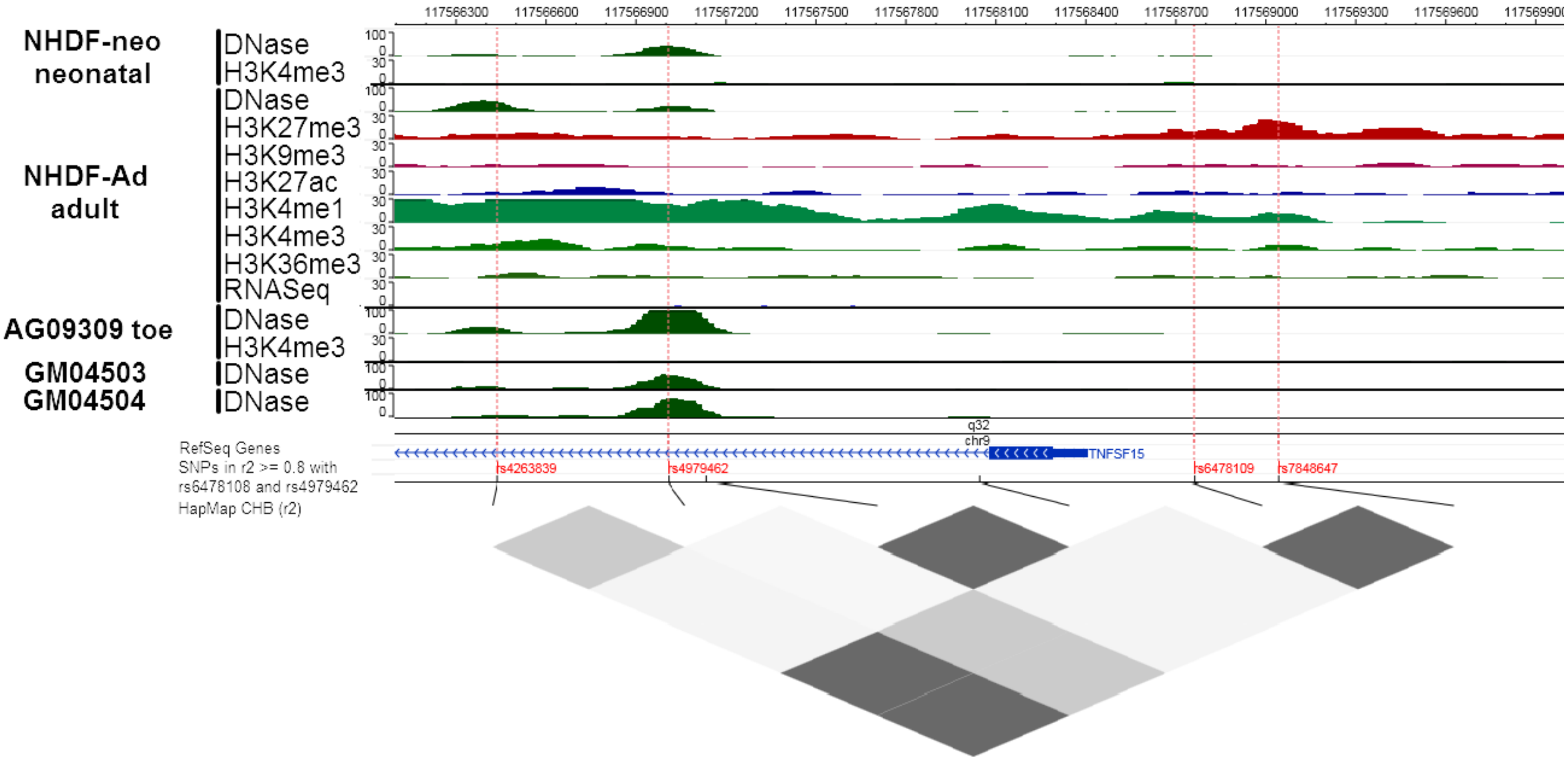

d) Other stromal and connective tissues/cells

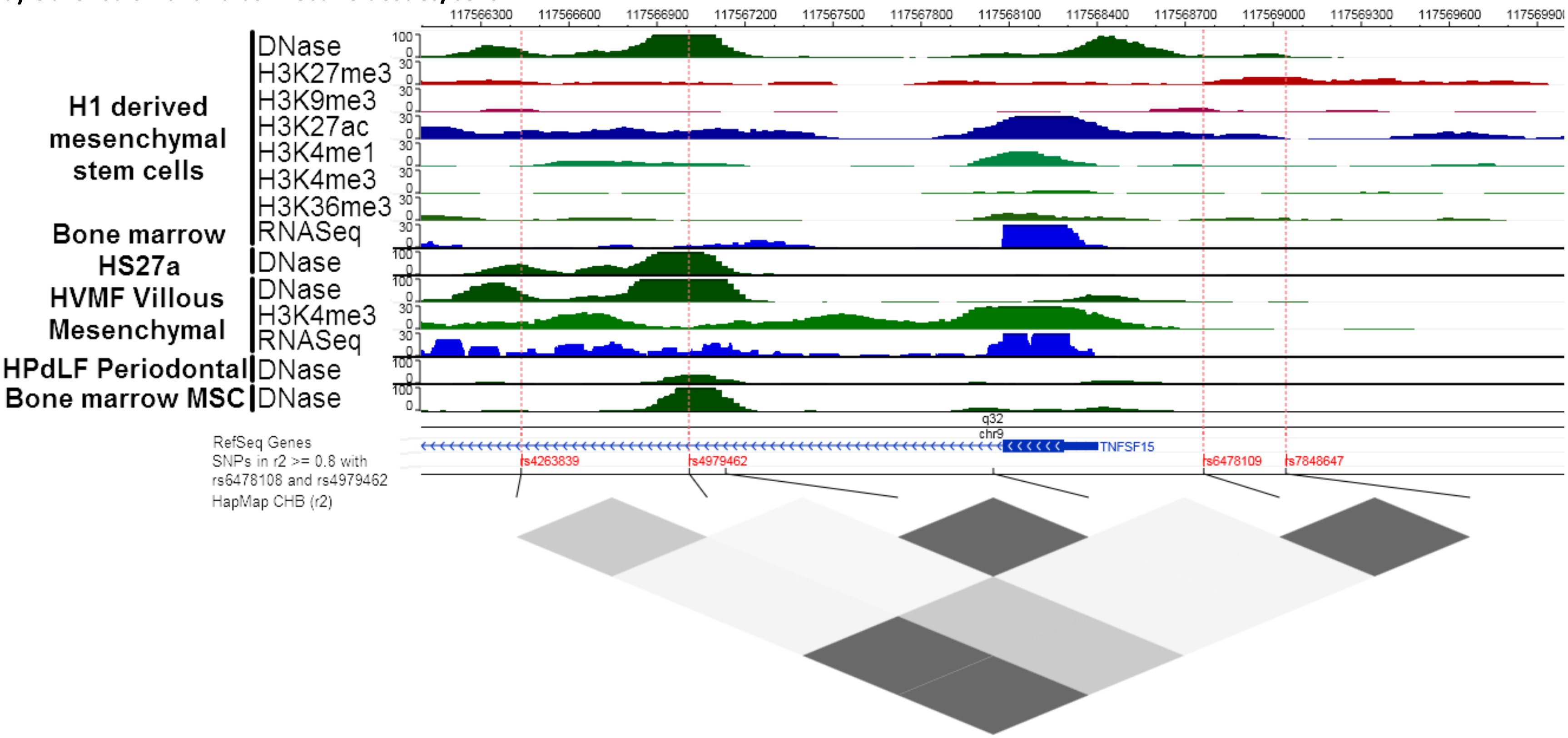

Supplementary Figure 7 Roadmap and ENCODE Annotation in Blood/Immune Cells

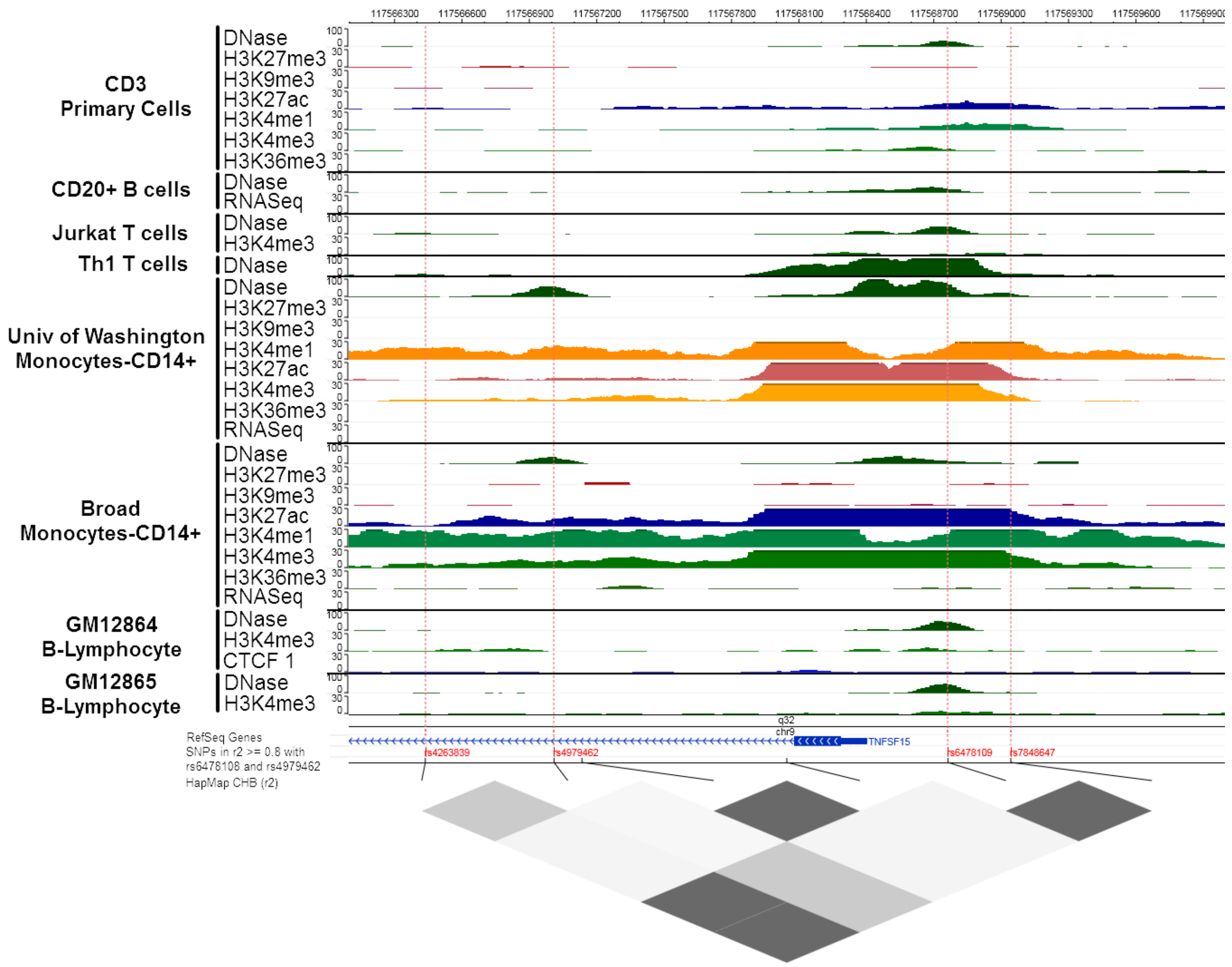

Supplementary Figure 8 Roadmap and ENCODE Annotation in Epithelial and Endothelial Tissues/Cells
a) Epithelial cancers

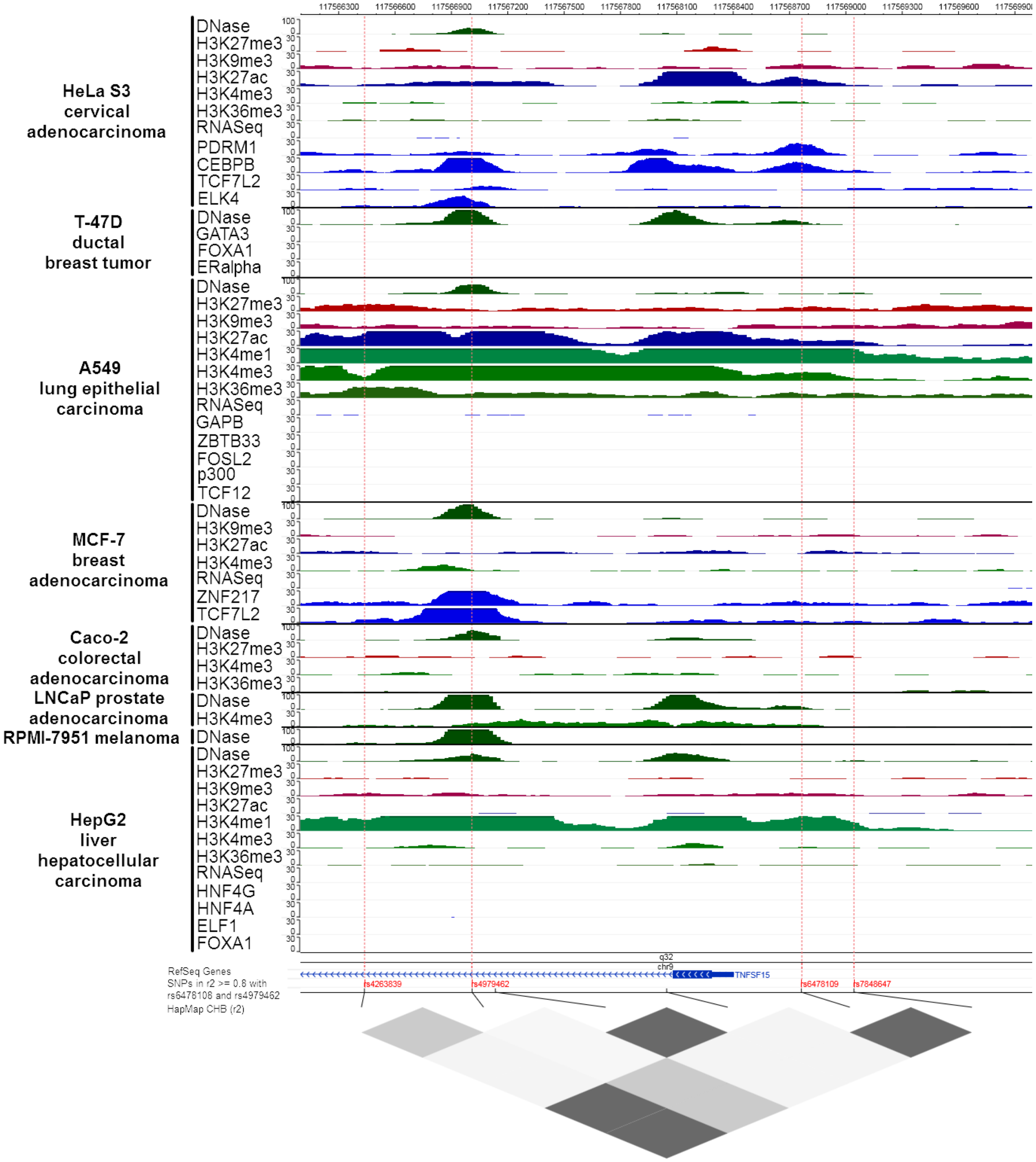

b) Microvascular endothelial cells

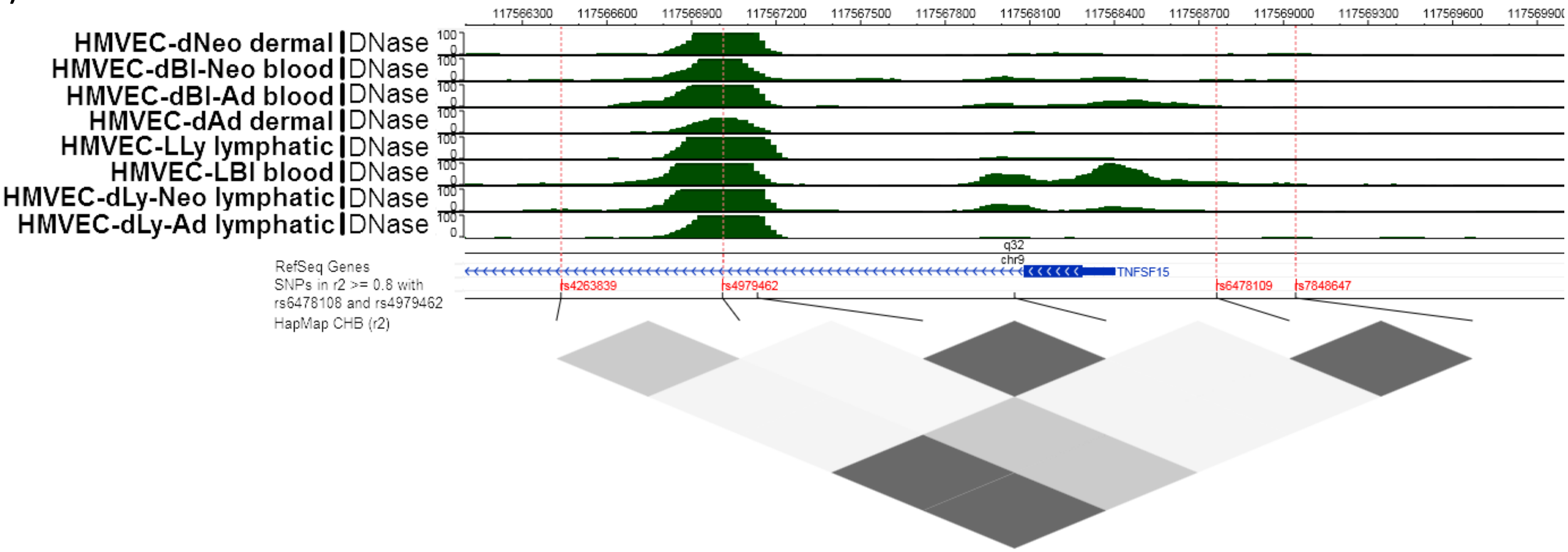

c) Other epithelial and endothelial cells

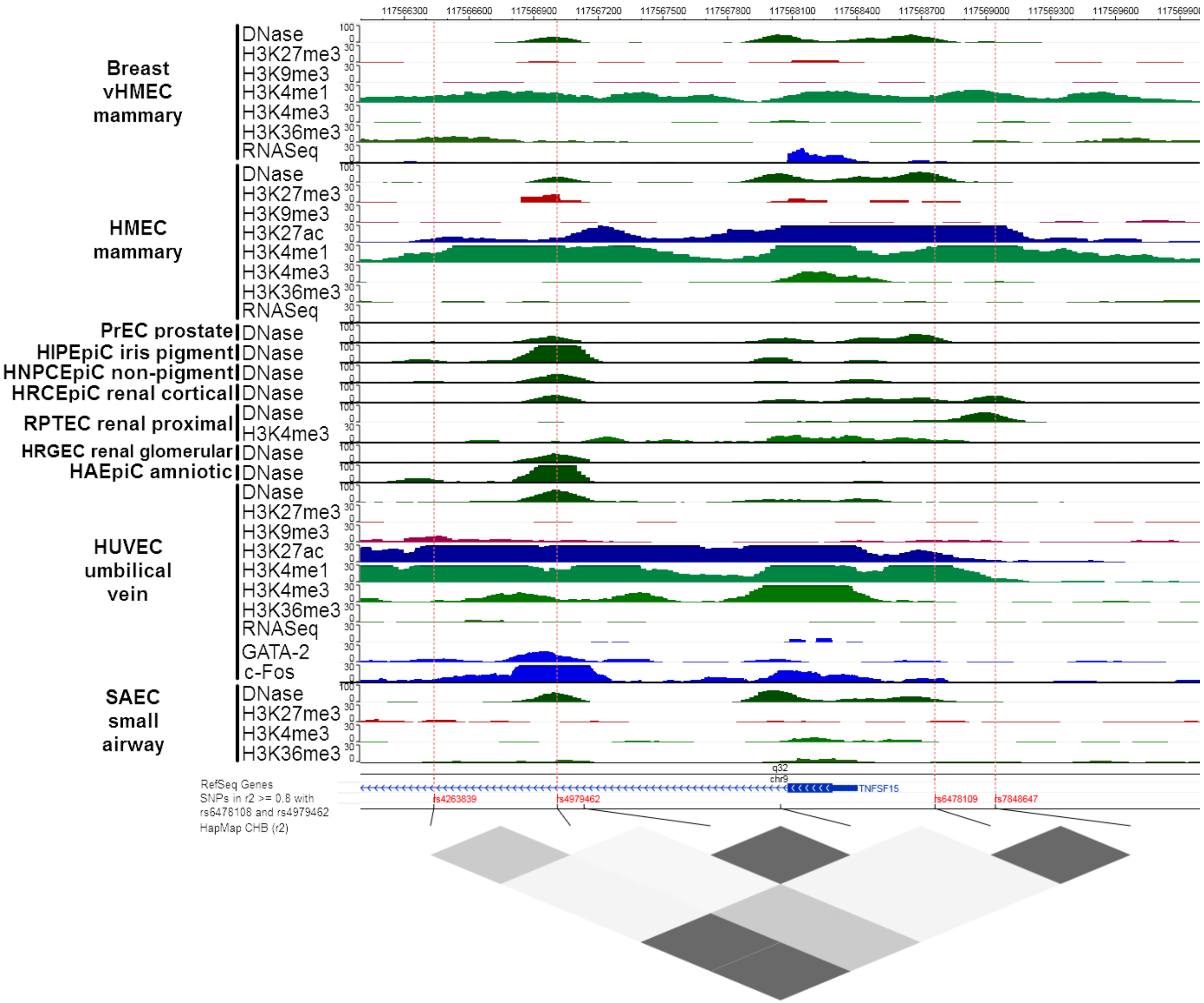

Supplementary Figure 9 Roadmap and ENCODE Annotation in Heart Tissues/Cells

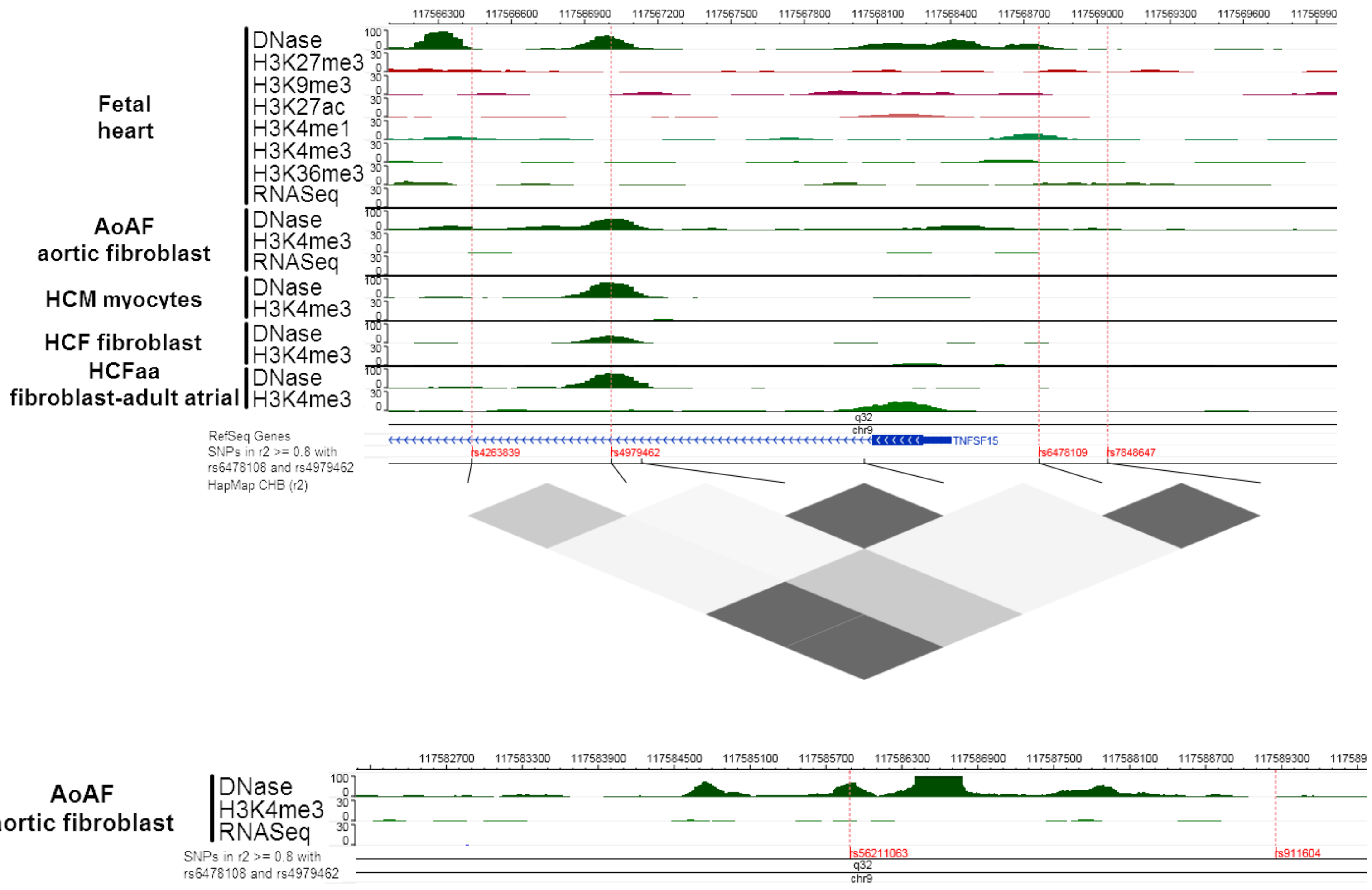

Supplementary Figure 10 Roadmap and ENCODE Annotation in Gastrointestinal Tissues/Cells

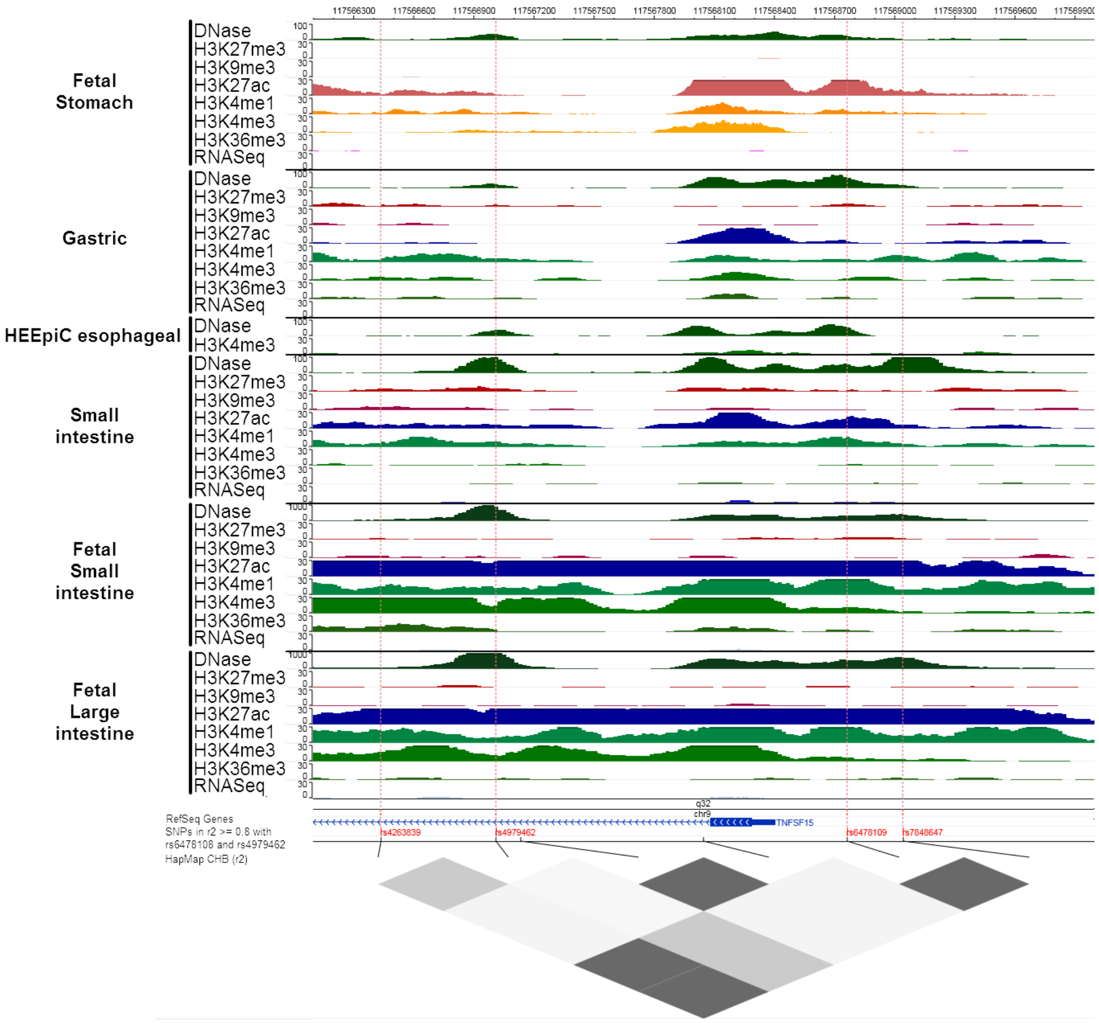

Supplementary Figure 11 Roadmap and ENCODE Annotation in Skeletal Muscle Tissues/Cells

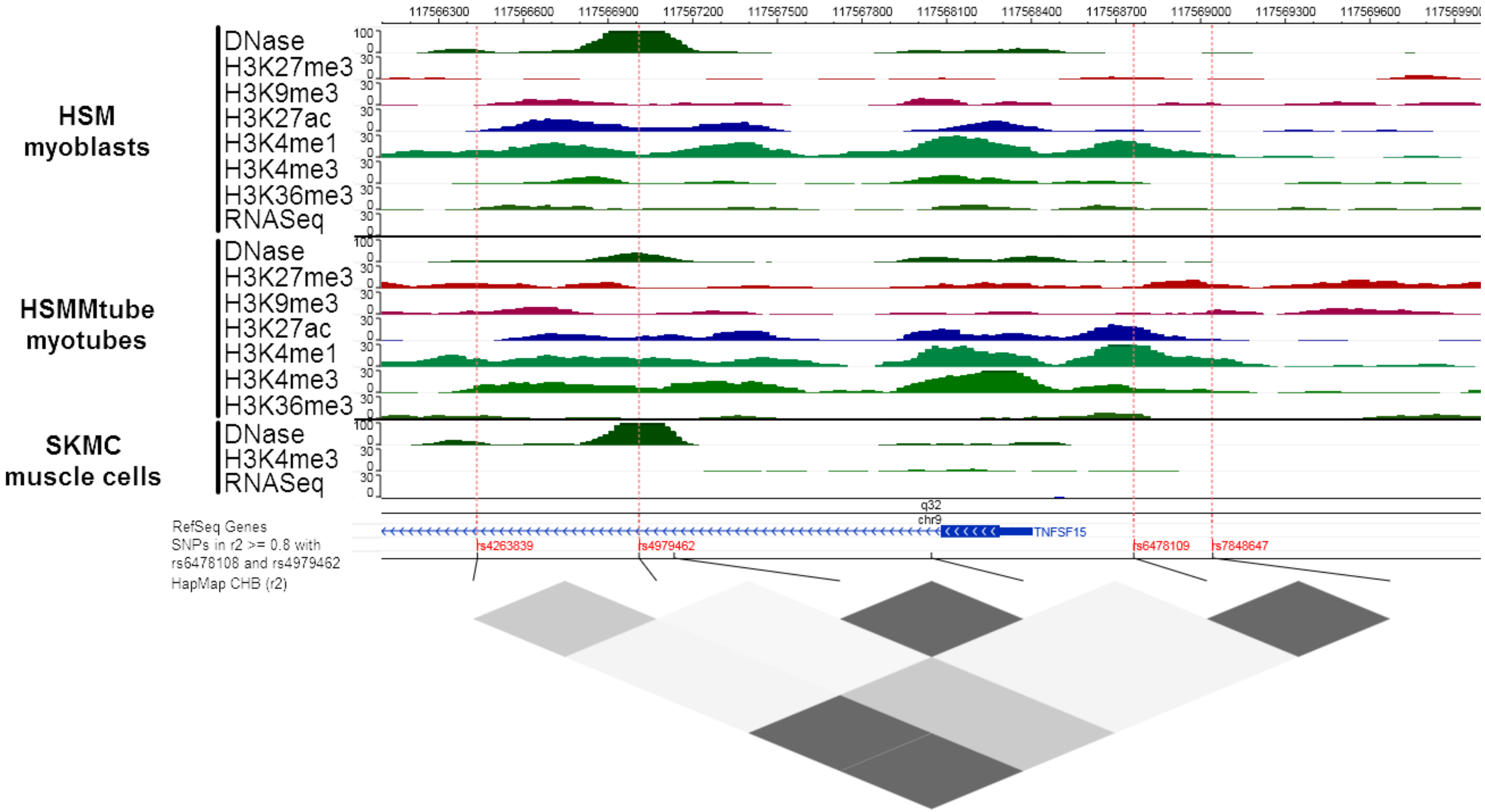

Supplementary Figure 12 Roadmap and ENCODE Annotation in Brain Tissues/Cells

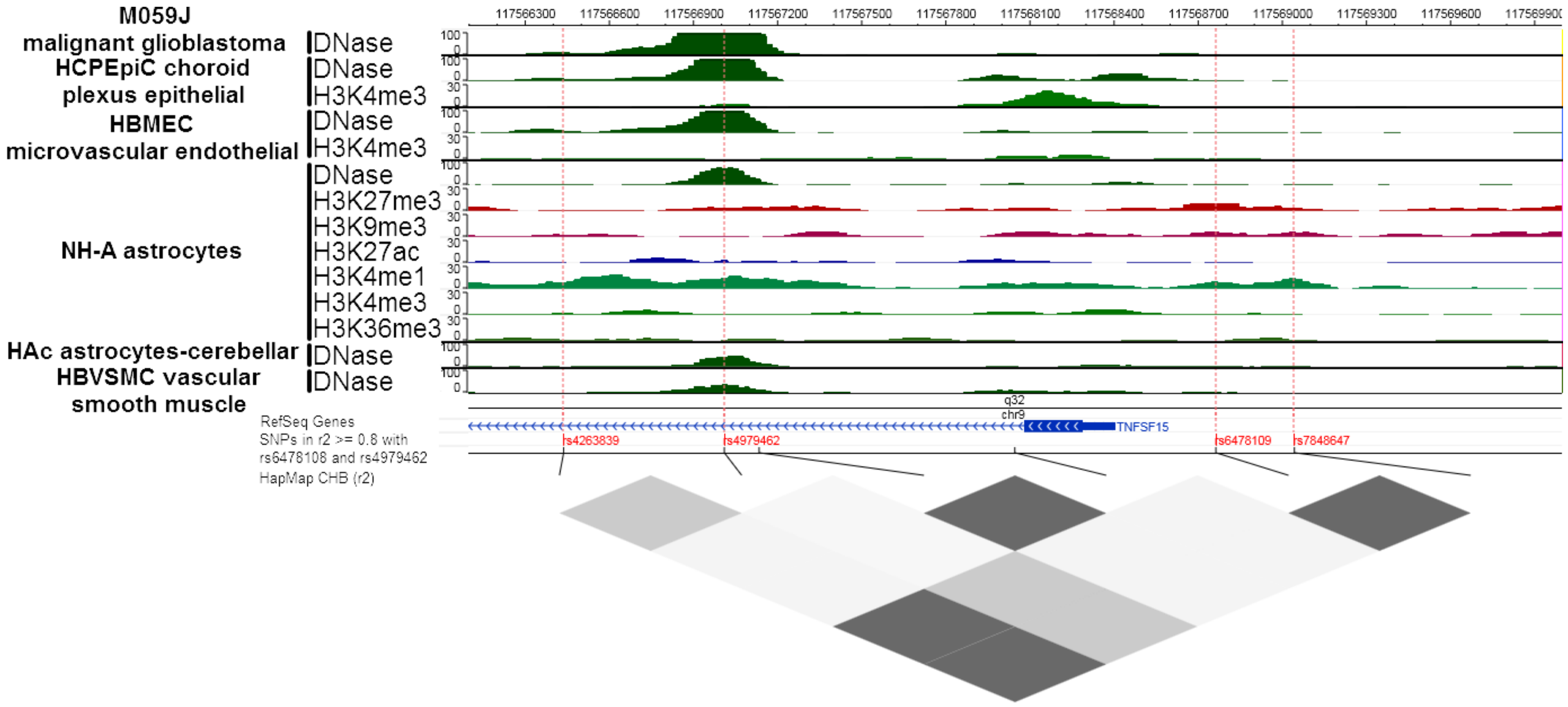

Supplementary Figure 13 Roadmap and ENCODE Annotation in Other Fetal Tissues/Cells

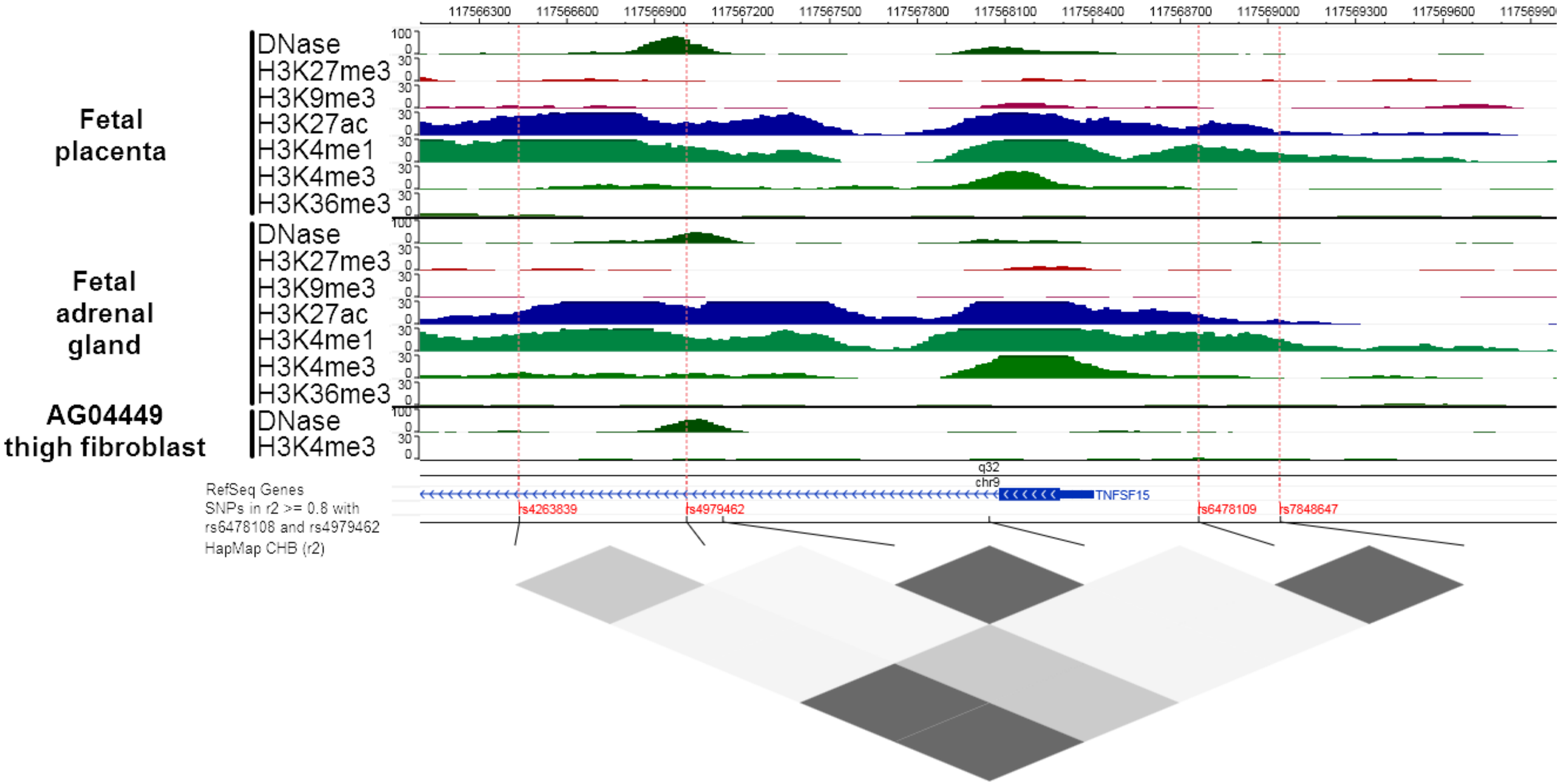

Supplement: Supplementary Information [file srep31429-s1.pdf]
